# Validation of Medicinal Leeches (*Hirudo medicinalis*) as a Non-invasive Blood Sampling Tool for Hematology and Biochemistry Profiling in Mammals

**DOI:** 10.3389/fvets.2022.831836

**Published:** 2022-02-04

**Authors:** Pavel Kvapil, Oldřich Tomášek, Eva Bártová, Mojca Harej, Marjan Kastelic, Tit Primožič, Nikola Kašpárková, Jožko Račnik

**Affiliations:** ^1^Ljubljana Zoo, Ljubljana, Slovenia; ^2^Department of Biology and Wildlife Diseases, Faculty of Veterinary Hygiene and Ecology, University of Veterinary Sciences Brno, Brno, Czechia; ^3^Institute of Vertebrate Biology, Czech Academy of Science, Brno, Czechia; ^4^Department of Botany and Zoology, Faculty of Science, Masaryk University, Brno, Czechia; ^5^Faculty of Veterinary Medicine, Institute of Poultry, Birds, Small Mammals and Reptiles, University of Ljubljana, Ljubljana, Slovenia

**Keywords:** hematology, biochemistry, non-invasive blood sampling, medicinal leech, *Hirudo medicinalis*

## Abstract

Blood sampling is a challenging procedure in many captive animals. Although manual restraint or anesthesia are usually possible, they entail intense stress and a high risk of injuries or organ failure. Blood sampling using medicinal leeches (*Hirudo medicinalis*) represents a promising non-invasive alternative to venipuncture; however, leech blood meal was to date used only for qualitative analyses such as genetic or serological screenings. Hence, the aim of this study was to evaluate the suitability of the leech blood sampling method for quantification of hematological and biochemical parameters. Medicinal leeches were manually applied on 67 zoo animals of eleven species, and control blood samples were obtained by venipuncture of the jugular vein. The leeches drew up to 20 ml of blood in 20 to 55 min. Although most hematological and biochemical parameters were significantly altered in leech-derived samples, their values showed strong (*r* = 0.62–0.79; 10/24 parameters) to very strong (*r* > 0.8; 13/24 parameters) correlations with venipuncture in all blood parameters, except for sodium (*r* = 0.39). As the parameter alterations and correlations were similar among species, simple cross-species regression formulas were sufficient to correct the alterations, thereby ensuring good repeatability between leeches and venipuncture in most parameters. Our data thus suggest that medicinal leeches can be used as a reliable non-invasive and stress-reducing alternative to standard venipuncture, even for quantitative assays. This opens new opportunities for a significant improvement to animal welfare in zoological gardens, conservation programmes, and ecophysiological research, where quantification of blood parameters is often needed.

## Introduction

Veterinary health assessment of animals in zoological collections or in semi-wild and wild conditions requires collection of blood samples for variety of qualitative and quantitative analyses, such as hematological, biochemical, genetic and endocrinological profiling, or screening of pathogenes causing infectious diseases. However, blood sampling is a challenging clinical procedure in such conditions, with anesthesia needed in most cases. Manual restraint is possible in some species, but it carries a high risk of injuries and intense stress for sampled animals. Therefore, non-invasive blood sampling is a promising tool for blood sample collection with a potential to substantially improve animal welfare in zoos, conservation programmes, and ecophysiological research in animal populations (1–5).

The applicability of kissing bugs—blood-sucking insects from the hemipteran subfamily Triatominae—for non-invasive blood sampling has previously been described (2, 6–11). *Dipetalogaster maxima* was the most common kissing bug species used due to its large size and therefore its capacity for extracting up to 1 ml of blood. Blood samples obtained by this method were used for measuring hormone levels, collecting genetic material, sexing, monitoring infectious diseases and measuring hematological and biochemical parameters (2, 6–10). However, kissing bugs are not commercially available and their breeding and maintenance are rather difficult. Another drawback is that they are nocturnal feeders and, hence, are highly sensitive to disturbance during feeding and usually only parasitise on motionless animals, ideally during the night. Other shortcomings include a narrow range of environmental temperatures acceptable for feeding and a relatively small blood volume that can be collected. Moreover, only a limited number of hematological and biochemical parameters can reliably be measured in their blood meal (2, 10).

Medicinal leeches (*Hirudo medicinalis*) from the family Hirudinidae are a potentially promising alternative because they are commercially available, not easily disturbed by the host animals, feed at much wider range of temperatures, and can yield much larger blood volumes. Medicinal leeches have been used in the treatment of many diseases for thousands of years (12) as bloodletting was a medical procedure believed to be a cure for anything from headache to gout (13). Nowadays, medical applications of leeches include, for example, treatments of osteoarthritis and venous congestion in plastic surgery, or various applications in alternative medicine (14–16). In animal science, samples extracted from wild caught leeches have been used as a source of viral DNA and RNA from wild vertebrates for biology studies (17). Moreover, the use of leech-derived blood samples for serological screening of zoo animals has recently been reported (4). However, to our knowledge, leech-derived blood samples have never been used for quantitative assays, such as screening of hematological and biochemical parameters, in either humans or any animal species.

To start filling this gap, the aim of our study was to evaluate the usability of medicinal leeches for non-invasive blood sampling in selected zoo animal species and the reliability of such blood samples used for hematology and biochemistry assessments. To this end, we compared hematological and biochemical values in the blood samples collected using leeches with those obtained by conventional venipuncture assessing the similarity of results of both methods. The similarity between two methods is best expressed as repeatability (5), which mostly depends on how strongly the results of both methods correlate and what is the mean difference between their results. For each blood parameter, we thus aimed to assess the strength of a correlation between both methods and examine potential alterations to the measured blood parameters, which could arise in the leech digestive tract (18). Nevertheless, potential alterations would not weaken reliability of the alternative blood sampling method if values obtained by both methods are strongly correlated; they only shift the means. We propose that, in such a case, correcting formulas could be used to correct altered values and ensure high repeatability between both blood sampling methods.

## Materials and Methods

### Zoo Animals

In total, 67 animals of 11 species (Table 1) were sampled: red-necked wallaby (*Macropus rufogriseus, n* = 1), European rabbit (*Oryctolagus cuniculus, n* = 29), brown rat (*Rattus norvegicus, n* = 1), Patagonian cavy (*Dolichotis patagonum, n* = 1), horse (*Equus caballus, n* = 3), Chapman's zebra (*Equus quagga chapmani, n* = 1), alpaca (*Vicugna pacos, n* = 8), Alpine ibex (*Capra ibex, n* = 7), domestic goat (*Capra aegagrus hircus, n* = 6), domestic sheep (*Ovis aries, n* = 9), and wild boar (*Sus scrofa, n* = 1). All animals were housed in the zoological collection at the Ljubljana Zoo (Slovenia) from their birth. The zebra was a 1-year-old intact male, and blood was sampled during anesthesia prior to transportation to another zoo. Blood samples from all other animals were collected during regular routine health checks. Blood samples from all animals were collected according to the standard operating protocols implemented during routine procedures as part of preventive health care.

**Table 1:** Characteristics of zoo animals used and leech sucking time.

**No**.	**English name**	**Scientific name**	**Age (y; m)**	**Sex** **(M=male, F=female)**	**Sucking time (min)**
1	domestic goat	*Capra aegagrus hircus*	11 y 6 m	F	35
2	domestic goat	*Capra aegagrus hircus*	9 y 11 m	F	30
3	domestic goat	*Capra aegagrus hircus*	8 y 4 m	F	35
4	domestic goat	*Capra aegagrus hircus*	7 y 10 m	F	40
5	domestic goat	*Capra aegagrus hircus*	6 y 4 m	F	25
6	domestic goat	*Capra aegagrus hircus*	6 m	F	45
7	Alpine ibex	*Capra ibex*	12 y	F	25
8	Alpine ibex	*Capra ibex*	5 y	F	20
9	Alpine ibex	*Capra ibex*	2 y 11 m	F	30
10	Alpine ibex	*Capra ibex*	1 y	M	25
11	horse	*Equus caballus*	19 y 1 m	F	30
12	horse	*Equus caballus*	14 y 4 m	M	35
13	horse	*Equus caballus*	4 y 3 m	F	45
14	Chapman's zebra	*Equus quagga chapmani*	1 y	M	25
15	European rabbit	*Oryctolagus cuniculus*	4 m	M	25
16	European rabbit	*Oryctolagus cuniculus*	4 m	F	20
17	European rabbit	*Oryctolagus cuniculus*	4 m	F	25
18	European rabbit	*Oryctolagus cuniculus*	4 m	F	35
19	European rabbit	*Oryctolagus cuniculus*	4 m	M	25
20	European rabbit	*Oryctolagus cuniculus*	4 m	M	20
21	European rabbit	*Oryctolagus cuniculus*	4 m	F	25
22	sheep	*Ovis aries*	12 y 1 m	F	55
23	sheep	*Ovis aries*	9 y 4 m	F	30
24	sheep	*Ovis aries*	6 y 7 m	F	50
25	sheep	*Ovis aries*	6 y 5 m	F	35
26	sheep	*Ovis aries*	5 y 10 m	F	35
27	sheep	*Ovis aries*	5 y 7 m	F	30
28	sheep	*Ovis aries*	3 y 5 m	M	40
29	sheep	*Ovis aries*	2 y 4 m	F	50
30	sheep	*Ovis aries*	3 m	M	20
31	brown rat	*Rattus norvegicus*	5 m	F	40
32	alpaca	*Vicugna pacos*	19 y 2 m	F	35
33	alpaca	*Vicugna pacos*	8 y 1 m	F	45
34	alpaca	*Vicugna pacos*	7 y 6 m	F	35
35	alpaca	*Vicugna pacos*	6 y	F	35
36	alpaca	*Vicugna pacos*	1 y 11 m	F	40
37	alpaca	*Vicugna pacos*	1 y	F	35
38	alpaca	*Vicugna pacos*	5 y	M	40
39	European rabbit	*Oryctolagus cuniculus*	4 m	M	30
40	European rabbit	*Oryctolagus cuniculus*	4 m	M	25
41	European rabbit	*Oryctolagus cuniculus*	4 m	F	20
42	European rabbit	*Oryctolagus cuniculus*	4 m	M	20
43	European rabbit	*Oryctolagus cuniculus*	4 m	F	25
44	European rabbit	*Oryctolagus cuniculus*	4 m	M	30
45	European rabbit	*Oryctolagus cuniculus*	4 m	F	35
46	red-necked wallaby	*Macropus rufogriseus*	1 y	M	25
47	Patagonian cavy	*Dolichotis patagonum*	6 y	M	25
48	European rabbit	*Oryctolagus cuniculus*	4 m	M	20
49	European rabbit	*Oryctolagus cuniculus*	4 m	F	25
50	European rabbit	*Oryctolagus cuniculus*	4 m	M	30
51	European rabbit	*Oryctolagus cuniculus*	4 m	M	35
52	European rabbit	*Oryctolagus cuniculus*	4 m	M	20
53	European rabbit	*Oryctolagus cuniculus*	4 m	F	25
54	European rabbit	*Oryctolagus cuniculus*	4 m	M	30
55	European rabbit	*Oryctolagus cuniculus*	4 m	F	35
56	European rabbit	*Oryctolagus cuniculus*	4 m	F	25
57	European rabbit	*Oryctolagus cuniculus*	4 m	F	20
58	European rabbit	*Oryctolagus cuniculus*	4 m	M	30
59	European rabbit	*Oryctolagus cuniculus*	4 m	F	35
60	European rabbit	*Oryctolagus cuniculus*	4 m	F	20
61	European rabbit	*Oryctolagus cuniculus*	4 m	M	25
62	European rabbit	*Oryctolagus cuniculus*	4 m	F	25
63	wild boar	*Sus scrofa*	4 m	M	30
64	Alpine ibex	*Capra ibex*	13 y	F	30
65	Alpine ibex	*Capra ibex*	9 y 9 m	M	35
66	Alpine ibex	*Capra ibex*	7 y 11 m	M	20
67	alpaca	*Vicugna pacos*	3 y	F	40

### Blood Sampling

Medicinal leeches were obtained from a commercial source (Biopharm, Hendy, South Wales, UK) and were kept individually in clean water at a temperature of 10 °C. One to three medium-sized unfed leeches were manually applied on individual animals depending on their body weight (one leech on the animal up to 1 kg, two leeches in animals up to 10 kg and three for animals above 10 kg). Hairless or areas sparsely covered with fur were preferable locations for leech application and none of the animals were clipped. Puncture of the jugular vein was used to acquire control blood samples collected at the same time frame of 20 min in domestic goat, domestic sheep, horse, alpaca, alpine ibex, and red-necked wallaby. In Patagonian cavy, brown rat, wild boar, and European rabbit, blood was collected from *vena cava cranialis* immediately after leech spontaneously fell off. The time taken to obtain the blood was measured from the beginning of the active sucking movement until the leeches spontaneously fell off the animals. After the leeches fell off, blood from their blood meal was collected by 5 ml or 10 ml syringe with 21G needle. The collected blood was placed into a Microvette® 500 μl LH (Lithium Heparin, Sarstedt, Germany) and analyzed immediately.

### Hematological and Biochemical Analysis

All blood samples were tested in an in-house laboratory at the zoo using a point-of-care hematology analyser (HM5, Abaxis, USA) and a point-of-care biochemistry analyser (VS2, Abaxis, USA). Biochemistry panel included 14 parameters: total protein (TP), albumin (ALB), globulin (GLOB), alkaline phosphatase (ALP), alanine aminotrasferase (ALT), amylase (AMY), total bilirubin (TBIL), blood urea nitrogen (BUN), creatinine (CRE), glucose (GLU), calcium (CA), phosphorus (PHOS), sodium (NAT), kalium (KAL). Hematology panel included 13 parameters: white blood cell count (WBC), lymphocyte count (LYM) and proportion (LYM%), monocyte count (MON) and proportion (MON%), neutrophil count (NEU) and proportion (NEU%), red blood cell count (RBC), hemoglobin (HGB), haematocrit (HCT), mean cell volume (MCV), mean corpuscular hemoglobin (MCH), and mean corpuscular hemoglobin concentration (MCHC). In addition, sample haemolysis and lipaemia were measured, but not analyzed statistically. The samples from 63 animals were used for biochemical analysis (Supplementary Table 1) due to marked haemolysis in the samples from three ibexes and one alpaca, while all animals tested (*n* = 67) were suitable for hematological analysis (Supplementary Table 2).

### Statistical Analysis

The data analysis was performed in R 4.1.1 statistical environment (19). The overall effects of leech sampling on focal blood parameters were analyzed across species using Bayesian modeling because this approach allows using multiple observations per species while controlling for phylogenetic autocorrelation. Another advantage of Bayesian inference is that it does not need corrections for multiple testing (20–22). The model structure used to test the differences between the leech method and venipuncture was equivalent to the paired *t*-test. The analysis was controlled for species identity and phylogeny (23), an approach standardly used in phylogenetic comparative studies [e.g., (24, 25)]. The blood parameter values were standardized by subtracting the mean and dividing by species-specific standard deviations calculated from the parameter reference ranges. The only exceptions were the proportional variables (LYM%, MON%, NEU%), which were divided by standard deviations calculated from the data, since reference ranges were unavailable for many species. The advantage of standardization is that the magnitudes of standardized effects (i.e., the differences between the leech method and venipuncture) are directly comparable among parameters (blood parameters showing larger effects are more strongly affected by the leech method), which would not be possible with the original units. The support for an effect was considered to be significant if its 95% credible intervals did not contain zero (26). In addition to the cross-species analysis, a separate analysis was done in three species with the largest sample sizes (rabbit, sheep, and alpaca) to examine the potential species differences in the leech-induced alterations. The relative proporions of lymphocytes, monocyte, and neutropils were used in this analysis as their changes could help the interpretation whether the potential alterations to hematology result from blood meal dehydration or some more complex process such as host reaction to the leech parasitic feeding.

To examine the correlations between the venipuncture- and leech-derived parameter values and potential species differences in these correlations, standard linear models were used. Given that there was little support for species differences in leech-venipuncture correlations and in how the leech method altered blood parameter values, we subsequently analyzed overall correlations between venipuncture and leeches across species.

To obtain reliable values of measured blood parameters, thereby ensuring the applicability of leech-derived blood samples, it is necessary to resolve the alterations in blood parameters induced by leeches. We here examined the performance of correction formulas *L*_c_ = *a* + *b*× *L*, where *L* and *L*_c_ are raw and corrected blood parameter values obtained using leeches, and *a* and *b* are intercept and slope from a simple regression model predicting venipuncture-derived values from leech-derived values. The performance of the correction formulas was assessed by comparing repeatabilities of leech- and venipuncture-derived values from the same individual. In our study design, repeatability (*R*; values 0–1) expresses the similarity between measurements obtained by both methods from the same individual, with *R* = 0 meaning no similarity at all, *R* = 0.5 meaning 50% similarity, and *R* = 1 resulting if the measurements from the same individual are identical. The absolute lymphocyte, monocyte, and neutrophil counts were used in this analysis as these original measured parameters were considered to be more suitable for the application of the corrections, rather than the relative proportions calculated from two measured parameters (i.e., the cell count of interest and WBC). Additional details on statistical analysis are provided in the Supplementary Material.

## Results

Medicinal leeches were able to draw 3–20 ml of blood in 20–55 min. The ambient temperature during sampling was between 7 and 23 °C and the humidity ranged from 38 to 70%. Data on zoo animals and leech sucking times are shown in Table 1.

**Figure 1:**
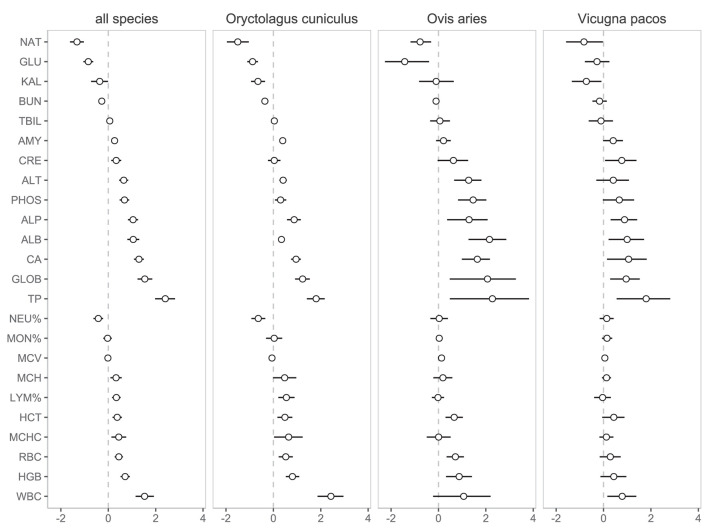
Standardized effect sizes of the leech-induced alterations to blood parameters. Presented are means from the Bayesian models with their 95% credible intervals. The effect is considered significant, if the credible interval does not contain zero (dashed lines). The effects are standardized to the species-specific standard deviations (i.e., effect size of one means that the values from the leech method differ from venipuncture by one standard deviation). The results in the leftmost panel were estimated from the data of all species using a Bayesian paired *t*-test controlling for species phylogenetic autocorrelation. NAT, sodium; GLU, glucose; KAL, potassium; BUN, urea nitrogen; TBIL, total bilirubin; AMY, amylase; CRE, creatinine; ALT, alanine aminotransferase; PHOS, phosphorus; ALP, alkaline phosphatase; ALB, albumin; CA, calcium; GLOB, globulin; TP, total protein; NEU, neutrophils; MON, monocytes; MCV, mean corpuscular volume; MCH, mean corpuscular hemoglobin; LYM, lymphocytes; HCT, haematocrit; MCHC, mean corpuscular hemoglobin concentration; RBC, red blood cells; HGB, hemoglobin; WBC, white blood cells.

The phylogenetically informed Bayesian *t*-test analysis of the complete data set showed that most of the hematological and biochemical parameters were altered by the leech sampling. The direction and strength of the effect varied greatly among parameters (Figure 1). Overall, most parameters were elevated in the leech-derived samples (*n* = 16; 67%). Only sodium, kalium, glucose, urea nitrogen, and the proportion of neutrophiles were reduced, whereas total bilirubin, MCV, and the proportion of monocytes showed no significant difference between the two sampling methods (Figure 1). Total protein was the most strongly affected parameter, with standardized effect size of 2.40 (95% credible interval: 1.98–2.82). Such a result implies that, relative to venipuncture, the total protein values in leech-derived samples were on average higher by 2.4 species-specific standard deviations, i.e., by more than a half (61%) of the species reference ranges, from which the standard deviations were estimated. Species-specific analyses in the rabbit, domestic sheep, and alpaca revealed very similar patterns of blood parameter alterations (Figure 1), suggesting that the effect of leech sampling is independent of the species being sampled.

The linear model, examining whether the slopes of relationships between leech- and venipuncture-based values vary among species, showed no differences in most parameters. Only phosphorus (*F* = 3.04; *p* = 0.02) and glucose (*F* = 2.90; *p* = 0.02) showed significantly different slopes among species. The lack of slope differences in most blood parameters among species provides further support for the hypothesis that the effect of leech sampling is similar across species.

**Figure 2:**
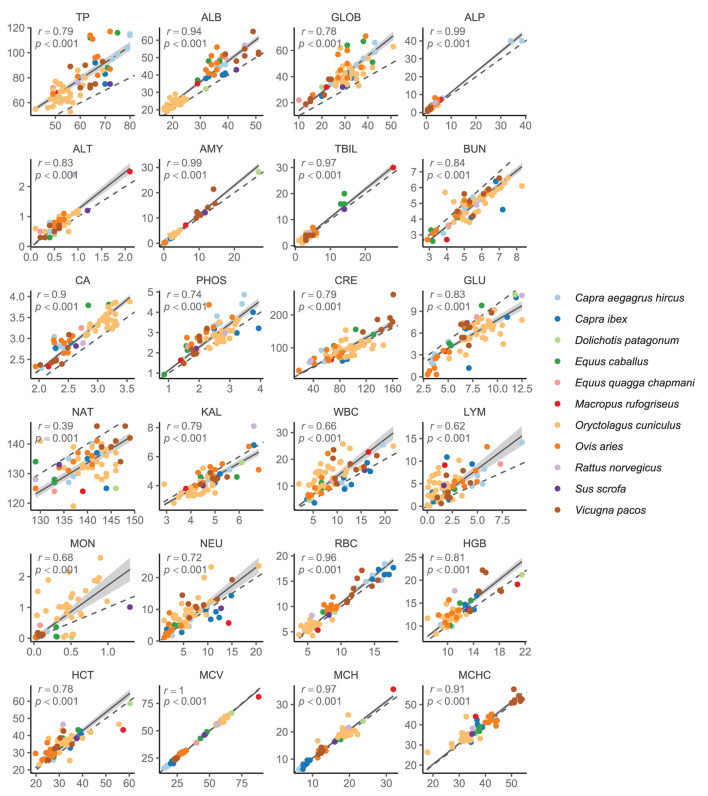
Relationships between venipuncture- (*x*-axis) and leech-derived (*y*-axis) values of measured blood parameters. Solid lines and shaded areas are regression lines and their 95% confidence intervals. Dashed lines depict 1:1 relationship. Shown are also Pearson's correlation coefficients (*r*) and their *p*-values. TP, total protein; ALB, albumin; GLOB, globulin; ALP, alkaline phosphatase; ALT, alanine aminotransferase; AMY, amylase; TBIL, total bilirubin; BUN, urea nitrogen; CA, calcium; PHOS, phosphorus; CRE, creatinine; GLU, glucose; NAT, sodium; KAL, potassium; WBC, white blood cells; LYM, lymphocytes; MON, monocytes; NEU, neutrophils; RBC, red blood cells; HGB, hemoglobin; HCT, haematocrit; MCV, mean corpuscular volume; MCH, mean corpuscular hemoglobin; MCHC, mean corpuscular hemoglobin concentration.

Consequently, we analyzed overall correlations between leech- and venipuncture-based values across species. The correlations were highly significant in all blood parameters (*p* ≥ 0.001; Figure 2). Out of 24 measured parameters, 23 (96%) showed a very strong (Pearson's *r* > 0.8; *n* = 13; 54%) or strong (*r* = 0.6–0.8; *n* = 10; 42%) correlation between leeches and venipuncture. Only sodium (*r* = 0.39) exhibited a weak correlation (Figure 2).

The strong correlations between leeches and venipuncture observed in most blood parameters examined in our study suggest that medicinal leeches represent a suitable method of blood sampling for hematology and clinical biochemistry. To ensure reliability of the obtained values, we applied regression formulas (Table 2) correcting the leech-induced alterations of analyzed blood parameters. As expected, such a correction greatly eliminated alterations of parameter value distributions (Supplementary Figure 1) and substantially increased repeatability between values obtained by leeches and venipuncture (Table 2).

**Table 2:** Correction formulas and repeatabilities between the leech method and venipuncture.

		**Correction formula**		**Repeatability**	
**Predictor**	**Panel**	**Intercept**	**Slope**	**Leech**	**Corrected**
TP	Biochemistry	26.620	0.424	0.14	0.77
ALB	Biochemistry	3.696	0.730	0.79	0.94
GLOB	Biochemistry	8.353	0.512	0.28	0.76
ALP	Biochemistry	−0.584	0.905	0.98	0.99
ALT	Biochemistry	0.124	0.625	0.71	0.82
AMY	Biochemistry	0.013	0.860	0.97	0.99
TBIL	Biochemistry	0.494	0.845	0.96	0.97
BUN	Biochemistry	0.644	0.986	0.71	0.83
CA	Biochemistry	0.037	0.876	0.63	0.90
PHOS	Biochemistry	0.628	0.642	0.59	0.71
CRE	Biochemistry	27.145	0.638	0.75	0.77
GLU	Biochemistry	2.816	0.788	0.66	0.82
NAT	Biochemistry	94.648	0.340	0.05	0.27
KAL	Biochemistry	1.789	0.667	0.72	0.77
WBC	Hematology	2.177	0.515	0.37	0.62
LYM	Hematology	0.539	0.382	0.26	0.57
MON	Hematology	0.123	0.305	0.42	0.64
NEU	Hematology	1.447	0.603	0.64	0.68
RBC	Hematology	−0.172	0.937	0.95	0.96
HGB	Hematology	1.597	0.761	0.68	0.79
HCT	Hematology	−0.189	0.934	0.63	0.66
MCV	Hematology	−1.188	1.029	1.00	1.00
MCH	Hematology	1.000	0.901	0.96	0.97
MCHC	Hematology	0.958	0.947	0.90	0.91

*Repeatability (R; values 0–1) expresses the similarity between measurements obtained by both methods from the same individual, with R = 0 meaning no similarity at all, R = 0.5 meaning 50% similarity, and R = 1 resulting if the measurements from the same individual are identical (i.e., 100% similarity). “Leech” column shows repeatabilities between venipuncture- and raw leech-derived values, whereas “Corrected” columns shows repeatabilities between venipuncture- and leech-derived values corrected using formulas L_c_ = a + b × L, where a and b are intercept and slopes, respectively, presented in the table and L are blood parameter values measured in leech-derived blood samples.*

*TP, total protein; ALB, albumin; GLOB, globulin; ALP, alkaline phosphatase; ALT, alanine aminotransferase; AMY, amylase; TBIL, total bilirubin; BUN, urea nitrogen; CA, calcium; PHOS, phosphorus; CRE, creatinine; GLU, glucose; NAT, sodium; KAL, potassium; WBC, white blood cells; LYM, lymphocytes; MON, monocytes; NEU, neutrophils; RBC, red blood cells; HGB, hemoglobin; HCT, haematocrit; MCV, mean corpuscular volume; MCH, mean corpuscular hemoglobin; MCHC, mean corpuscular hemoglobin concentration*.

## Discussion

Medicinal leeches represent a promising biological tool for non-invasive blood sampling of animals in zoos (4) or in semi-wild and wild conditions (17). However, blood obtained by this method has never been used for quantitative diagnostic procedures such as hematological and biochemical analysis. Here, we show that most of the biochemical and hematological parameters are strongly correlated between leech-derived blood samples and standard venipuncture, suggesting that medicinal leeches can indeed be used as a reliable blood sampling tool for hematological and biochemical health assessment. Yet, our results also showed that the blood parameters are systematically altered in leech-derived blood samples, which needs to be taken into consideration during their clinical interpretation.

Nevertheless, the alterations in measured blood parameters in leech-derived samples does not preclude the usability of such samples for hematological and biochemical health assessment. The most important quality in this regard is the strength of the correlation between an alternative method and venipuncture. When the correlation is strong, the alternative method can give reliable values despite parameter alterations, provided that an appropriate correction is applied, or reference ranges based on the alternative method are used. The correlations between the parameter values obtained by leeches and venipuncture were strong or very strong in most blood parameters measured, except for sodium. Such a result suggests that medicinal leeches can indeed be reliably used for hematological and biochemical health assessment.

Despite the strong correlations, the alterations in biochemistry and hematology parameters in samples obtained by leeches must be considered. Most of the parameters were elevated, with total protein being most strongly affected. Such a pattern could result from blood dehydration in the digestive system of leeches because they excrete the excess water content of the blood meal in order to obtain as many nutrients as possible (18). Sodium, glucose, and urea nitrogen were the only biochemical parameters showing reduction in leech-derived samples, with sodium and glucose showing the strongest effects. In the previous studies on hematophagous bugs *Rhodnius prolixus* and *Dipelogaster maxima*, the sodium value was also significantly lower in the bug-derived blood (2, 27). Reduction in sodium is most probably associated with blood dehydration in the digestive systems of leeches and bugs as sodium plays a major role in water balance (2, 18, 27). Glucose absorption in the gut and its usage in leech metabolic processes could possibly explain the reduction of glucose values in the samples obtained by leeches as medicinal leeches have only small reserves of glycogen (28).

WBC count was the most strongly affected parameter in the hematology panel. Its elevation in leech-derived samples may also be a result of blood dehydration. Alternative, but not mutually exclusive explanation, could be a mobilization of WBC by the host in response to injury and the presence of foreign peptides associated with leech parasitic feeding. The occurrence of changes in relative proportions of lymphocytes (elevated) and neutrophils (reduced) apparent in Figure 1 may provide support for the latter explanation, rather than for the simple effect of blood dehydration. Nevertheless, blood dehydration probably also plays a role, considering the changes in biochemistry and elevated absolute counts of all three main WBC types analyzed in our study (Figure 2). Interestingly, the opposite pattern has been observed in blood samples collected using hematophagous bugs *D. maxima* where WBC values were significantly lower than those collected by conventional venipuncture (2).

The parameter alterations observed in the three species with the highest sample sizes showed patterns that were similar to the overall cross-species pattern, as well as to each other. Furthermore, the leech-vena regression slopes did not differ among species in most parameters. Together with the strong overall correlations in the full data set, these results suggest that the effect of leech sampling does not differ between species. This implies that reliable values could be obtained using a single correction formula per parameter irrespective of the species. We provide such correction formulas based on the regressions of venipuncture-derived values on leech-derived values. A substantial improvement in repeatability between corrected leech-derived values and those obtained by venipuncture indicates reliability of such an approach since repeatability expresses the degree of similarity between the measurements obtained by both methods from the same individual.

All leeches in our study were collected and their blood meals were withdrawn immediately after the leeches fell off the animal. We believe that such an immediate blood sample processing should be possible in most sampling scenarios because the sampling should always be overseen to ensure the identity of the sampled animals, evaluate sampling success, and prevent leech loss after it falls off. In scenarios where the immediate blood sample processing is either impossible or inconvenient, the effect of the time period between the end of feeding and the sample processing should be examined and, if significant, it could be included in the correction formula.

Although a systematic analysis of factors affecting the success and duration of feeding was not the main focus of our study, our observations suggest that external conditions such as light/sun intensity, temperature, and vibrations during the manipulation of leeches affect both the success of blood withdrawal and the duration of feeding. Leeches usually come in the ready-to-feed state from the suppliers; however, lower willingness to feed observed in two batches suggests that the transport of the leeches may be an important factor affecting their viability. We further observed that leeches avoid feeding on direct sunlight and, consequently, the optimal conditions proved to be cloudy weather with temperature between 15 and 20 °C. Nevertheless, leeches are very hungry animals as their ecological strategy demands them to be and were able to feed in a relatively wide temperature range (7–23 °C) when shaded from direct sunlight. Importantly, leeches seemed to prefer feeding on calm animals rather than the ones stressed due to the manual restraint, which further supports their suitability for non-invasive blood sampling. Moreover, in our experience, the animals tolerate leeches better than kissing bugs and do not try to remove leeches in most cases.

One of the expected potential complications was prolonged bleeding from the site of the attachment after the leech fell off. Surprisingly, prolonged post-extraction bleeding was not observed in any of the 67 animals involved in this study and has stopped after a few minutes in most. The maximal bleeding time has been difficult to evaluate as all animals have been released immediately after sample collection and bleeding has been only observed from distance. Unfortunately, to the best of our knowledge, the information on bleeding times following leech feeding is lacking in mammals other than human. The only comparative study reporting bleeding times among taxa only included human and several species of fish and amphibians (29). In general, humans show much longer bleeding times (hours to days) than fish and amphibians (several minutes). Whether the human bleeding times are standard or rather exceptional among mammals is unclear given the lack of data from other mammalian species. Our observations suggest the latter may be true but further studies are need. Leech feeding also caused no other clinical abnormalities or wounds, which would prevent the exhibition of animals in the zoo collection for aesthetic reasons.

All leeches in our study have been applied on either manually or chemically restrained animals, which was the only way how venipuncture could be performed at the same time to directly compare both methods. Nevertheless, to successfully apply the leech method for non-invasive blood sampling, methods of leech application to non-restrained fully conscious animals need to be developed. Although not used in this study, we have developed a method using a wooden platform with holes for bowls that contain leeches and are covered with Parafilm M (Bemis, USA) sealing film. The wooden platform is placed on the spot where the target animal usually rests and after the animal lies down on the platform, the leeches bite through the membrane and start feeding on the animal. In our experience, closing an animal in a box with leeches placed next to them also works in some species (e.g., rabbit or porcupine) and is still safer and less stressful than venipuncture under manual restraint or anesthesia. We believe that many alternative methods could be developed that would be suitable for various species and conditions.

## Conclusion

Non-invasive blood sampling methods that do not require animal restraint, thus having the potential to substantially improve welfare in sampled animals, are of a great interest in zoo medicine, ecological research, and conservation physiology (1, 3–5). Medicinal leeches have previously been demonstrated to be a suitable source of blood samples for molecular and serological screenings (4, 17). Taking it a step further, we here show that blood samples obtained using medicinal leeches are also suitable for quantitative assays such as hematology and biochemistry profiling. Our data from 11 mammal species suggest that the leech-induced alterations to the blood parameters analyzed are not species-specific and can be to a large extent corrected by generally applicable regression formulas that we provide. Nonetheless, the strength of correlation between leech- and venipuncture-derived values (and hence the level of error associated with the leech method) varies among parameters, and this needs to be considered when interpreting the obtained profiles. Future studies are also advisable to examine whether the accuracy of estimated blood parameters can further be increased by species-specific correction formulas. Alternatively, reference ranges for blood samples collected using leeches could be determined once sufficient sample sizes are available for species of interest. Besides, further validation is necessary to determine suitable methods and body sites of leech application and potential effects of environmental conditions (5). Our results suggest that such efforts are worth undertaking and, when validated, medicinal leeches can become a valuable addition to the blood sampling toolkit in veterinary medicine, ecological research, and conservation.

## Data Availability Statement

The original contributions presented in the study are included in the article/Supplementary Material, further inquiries can be directed to the corresponding authors.

## Ethics Statement

All samples were collected as a secondary interest by zoo personal during clinical procedures, surgeries, and annual health check-ups. All procedures involving animals were approved by the National Ethical Committee and the Administration of the Republic of Slovenia for Food Safety, Veterinary and Plant Protection. Animal care and treatment were conducted in accordance with the institutional guidelines and international laws and policies (Directive 2010/63/EU on the protection of animals used for scientific purposes).

## Author Contributions

PK: conceptualization, investigation, data curation, writing-original draft preparation, and writing-review and editing. OT: conceptualization, formal analyses, visualization, writing-original draft preparation, writing-review and editing, and supervision. EB: conceptualization, methodology, resources, writing-original draft preparation, writing-review and editing, supervision, and funding acquisition. MK: investigation, data curation, and project administration. JR: conceptualization, writing-review and editing, methodology, supervision, and validation. MH, TP, and NK: investigation, data curation, and writing-review and editing. All authors read and approved the final version of the manuscript.

## Funding

The study was financially supported from the internal grant of University of Veterinary Sciences Brno (FVHE/Literák/ITA2020) and the Czech Science Foundation (GA21-22160S to OT).

## Conflict of Interest

The authors declare that the research was conducted in the absence of any commercial or financial relationships that could be construed as a potential conflict of interest.

## Publisher's Note

All claims expressed in this article are solely those of the authors and do not necessarily represent those of their affiliated organizations, or those of the publisher, the editors and the reviewers. Any product that may be evaluated in this article, or claim that may be made by its manufacturer, is not guaranteed or endorsed by the publisher.

## Abbreviations

ALB: albumin
ALP: alkaline phosphatase
ALT: alanine aminotransferase
AMY: amylase
BUN: urea nitrogen
CA: calcium
CRE: creatinine
GLOB: globulin
GLU: glucose
HCT: haematocrit
HGB: hemoglobin
KAL: potassium
LYM: lymphocytes
MCH: mean corpuscular hemoglobin
MCHC: mean corpuscular hemoglobin concentration
MCV: mean corpuscular volume
MON: monocytes
NAT: sodium
NEU: neutrophils
PHOS: phosphorus
RBC: red blood cells
TBIL: total bilirubin
TP: total protein
WBC: white blood cells.
