# Supplementary material for: Validation of Medicinal Leeches (Hirudo medicinalis) as a Non-invasive Blood Sampling Tool for Hematology and Biochemistry Profiling in Mammals
Source: Front Vet Sci. 2022 Feb 4;9:831836. doi: 10.3389/fvets.2022.831836 (PMC8855102; doi:10.3389/fvets.2022.831836)
Supplement: Supplementary file 1 [file Data_Sheet_1.DOCX]

Supplementary Material

# Suplementary Methods

## Statistical Analysis

The data analysis was performed in R 4.1.1 statistical environment (1). The overall effects of leech sampling on focal blood parameters was analysed across species using Bayesian modelling based on the Stan language as implemented in the brms R package (2). Bayesian approach was chosen for this analysis because it allows using multiple observations per species in phylogenetically informed models. In addition, Bayesian inference does not need corrections for multiple testing because the probability estimate of an effect of interest is regularised by the prior and, unlike the frequentist framework, Bayesian inference does not make assumptions about the distribution of *p* values in theoretical repetitions of an experiment (3; 4; 5). Each model included values of a focal blood parameter as a response variable, the sampling method (0 = venipuncture, 1 = leeches) as a predictor, and individual ID as a random grouping variable (varying intercept). Such a model structure is equivalent to the paired *t*-test. Model further included species ID and phylogeny as additional random effects to control for non-independence of observations from the same species while factoring in species phylogenetic relationships (6). Such an approach is standardly used in phylogenetic comparative studies (7; 8). For the eleven species included in our study, we downloaded 1000 phylogenetic trees from www.VertLife.org (9) and generated a maximum clade credibility tree using R package phangorn (10). The blood parameter values were standardised (*z*-transformed) by subtracting the mean and dividing by species-specific standard deviation (SD) calculated from the parameter reference ranges (RR) obtained from the ZIMS database according to the formula SD = RR / (2 × 1.96). The only exceptions were the proportional variables (LYM%, MON%, NEU%), which were normalised using logit transformation, and subsequently divided by standard deviation calculated from the data, since reference ranges were unavailable for many species. A commonly used weakly informative Gaussian prior (*μ* = 0, *σ* = 1) was used for the effects and default brms priors based on Student’s *t*‑distribution were used for the fixed and varying intercepts (11). In addition, within-species models without the random effects of species ID and phylogeny were separately fitted in the three species with the largest sample sizes (rabbit, sheep, and alpaca) to examine the potential species differences in the leech-induced alterations. The models ran in four chains, each with 11,000 iterations, with warm-up set to 1000 iterations and thinning to 10. Potential scale reduction factor was ~1.00 in all cases, indicating good model convergence (12).

The resulting estimates are presented as posterior means together with their equal-tailed 95% credible intervals based on quantiles (2). In the Bayesian framework, the 95% credible interval represents a range of values that, given the prior and observed data, contain the true effect value with 95% probability. The support for an effect was considered to be significant if 95% credible intervals did not contain zero (13).

To examine the correlations between the venipuncture- and leech-derived parameter values and potential species differences in these correlations, the linear model was fitted, including leech-derived values as a response variable and venipunture-derived values, species, and venipunture × species interaction as predictors. Interaction effects with *p* ≤ 0.05 were regarded as a significant support for species differences in the slope of venipuncture-leech relationship. Given that there was little support for species differences in leech-venipuncture correlations and in how leech sampling altered blood parameter values, we subsequently analysed overall Pearson’s correlations between venipuncture and leeches across species.

To obtain reliable values of focal parameters and ensure the applicability of leech-derived blood samples, it is necessary to resolve the alterations in blood parameters induced by leeches. We here examined the performance of correction formulas *L*_c_ = *a* + *b* × *L*, where *L* and *L*_c_ are raw and corrected blood parameter values obtained using leeches, and *a* and *b* are intercept and slope from the regression model of venipuncture-derived values (*V*) on leech-derived values: *V* = *a* + *b* × *L* + *ε*, where *ε* is a Gaussian error. The performance of the correction formulas was assessed by comparing repeatabilities of leech- and venipuncture-derived values from the same individual. Repeatabilities were calculated as intra-class correlation coeffcients from the mixed-effects models with individual ID as a random grouping variable (varying intercept) (14). In these models, values from venipuncture together with either raw or corrected leech-derived values were fitted as a response variable. Repeatability (*R*; values 0–1) from such a model structure expresses the similarity between measurements obtained by both methods from the same individual, with *R* = 0 meaning no similarity at all, and *R* = 1 resulting if the measurements from the same individual are identical.

# 1.2 References

1. R Core Team. *R: A language and environment for statistical computing.* Vienna, Austria: R Foundation for Statistical Computing. http://www.R-project.org/ (2021). Available at: http://www.R-project.org/
2. Bürkner P-C. brms: an R package for Bayesian multilevel models using Stan. *J Stat Soft.* (2017) 80:1–28. doi:10.18637/jss.v080.i01
3. Berry DA, Hochberg Y. Bayesian perspectives on multiple comparisons. *J Stat Plan Inference.* (1999) 82:215–227. doi:10.1016/S0378-3758(99)00044-0
4. Gelman A, Hill J, Yajima M. Why we (usually) don’t have to worry about multiple comparisons. *J Res Educ Eff..* (2012) 5:189–211. doi:10.1080/19345747.2011.618213
5. Sjölander A, Vansteelandt S. Frequentist versus Bayesian approaches to multiple testing. *Eur J Epidemiol.* (2019) 34:809–21. doi:10.1007/s10654-019-00517-2
6. Garamszegi LZ. “Uncertainties due to within-species variation in comparative studies: Measurement Errors and Statistical Weights,” in *Modern Phylogenetic Comparative Methods and Their Application in Evolutionary Biology*, ed. L. Z. Garamszegi (Heidelberg: Springer), 157–99.
7. Tomasek O, Bobek L, Kralova T, Adamkova M, Albrecht T. Fuel for the pace of life: baseline blood glucose concentration co-evolves with life-history traits in songbirds. *Funct Ecol.* (2019) 33:239–49. doi:10.1111/1365-2435.13238
8. Kumar SA, Albrecht T, Kauzál O, Tomášek O. No evidence for trade-offs between lifespan, fecundity and basal metabolic rate mediated by liver fatty acid composition in birds. *Front Cell Dev Biol.* (2021) 9:638501. doi:10.3389/fcell.2021.638501
9. Upham NS, Esselstyn JA, Jetz W. Inferring the mammal tree: Species-level sets of phylogenies for questions in ecology, evolution, and conservation. *PLOS Biology.* (2019) 17:e3000494. doi:10.1371/journal.pbio.3000494
10. Schliep KP. phangorn: phylogenetic analysis in R. *Bioinformatics.* (2011) 27:592–3. doi:10.1093/bioinformatics/btq706
11. Bürkner P-C. Advanced Bayesian Multilevel Modeling with the R Package brms. *R J.* (2018) 10:395. doi:10.32614/RJ-2018-017
12. Gelman A, Rubin DB. Inference from iterative simulation using multiple sequences. *Statist Sci.* (1992) 7:457–72. doi:10.1214/ss/1177011136
13. Hespanhol L, Vallio CS, Costa LM, Saragiotto BT. Understanding and interpreting confidence and credible intervals around effect estimates. *Braz J Phys Ther.* (2019) 23:290–301. doi:10.1016/j.bjpt.2018.12.006
14. Nakagawa S, Schielzeth H. Repeatability for Gaussian and non-Gaussian data: a practical guide for biologists. *Biol Rev.* (2010) 85:935–956. doi:10.1111/j.1469-185X.2010.00141.x

# Supplementary Results

# Supplementary Table 1 Results of 14 biochemistry parameters and sample haemolysis and lipaemia in individual zoo animals (n = 63) in blood samples collected by leeches and venipuncture. Total protein (TP, g/L), albumin (ALB, g/L), globulin (GLOB, g/L), alkaline phosphatase (ALP, µkat/L), alanine aminotrasferase (ALT, µkat/L), amylase (AMY, µkat/L), total bilirubin (TBIL, µmol/L), blood urea nitrogen (BUN, mmol/L), creatinine (CRE, µmol/L), glucose (GLU, mmol/L), calcium (CA, mmol/L), phosphorus (PHOS, mmol/L), sodium (NAT, mmol/L), and potassium (KAL, mmol/L), haemolysis (HEM), lipemia (LIP), not available (NA), leech sample (Leech), venipuncture sample (Vena).

| No. | Species | Method | TP | ALB | GLOB | ALP | ALT | AMY | TBIL | BUN | CRE | GLU | CA | PHOS | NAT | KAL | HEM | LIP |
| --- | --- | --- | --- | --- | --- | --- | --- | --- | --- | --- | --- | --- | --- | --- | --- | --- | --- | --- |
| 1 | *Capra aegagrus hircus* | Leech | 114 | 49 | 66 | 8.0 | 0.8 | 0.2 | NA | 4.9 | NA | 2.0 | 3.04 | 3.39 | 132 | NA | 3 | 0 |
| 1 | *Capra aegagrus hircus* | Vena | 80 | 37 | 43 | 6.6 | 0.4 | 0.5 | 4 | 5.0 | 75 | 3.3 | 2.26 | 2.57 | 140 | 4.6 | 0 | 0 |
| 2 | *Capra aegagrus hircus* | Leech | 104 | 45 | 59 | 40.0 | 0.5 | 1.0 | 4 | 5.4 | 91 | 2.5 | 2.81 | 3.45 | 140 | 4.3 | 1 | 0 |
| 2 | *Capra aegagrus hircus* | Vena | 79 | 37 | 43 | 34.0 | 0.4 | 0.8 | 4 | 5.5 | 76 | 3.5 | 2.38 | 2.65 | 141 | 4.4 | 0 | 0 |
| 3 | *Capra aegagrus hircus* | Leech | 99 | 45 | 54 | 40.0 | 0.7 | 0.5 | 4 | 3.1 | 55 | 1.7 | 2.77 | 3.81 | 129 | 4.7 | 2 | 0 |
| 3 | *Capra aegagrus hircus* | Vena | 75 | 36 | 38 | 38.5 | 0.4 | 0.5 | 4 | 3.8 | 34 | 3.5 | 2.43 | 2.58 | 137 | 4.7 | 0 | 0 |
| 4 | *Capra aegagrus hircus* | Leech | 115 | 49 | 66 | 1.1 | 0.7 | 0.8 | 4 | 4.7 | 92 | 1.8 | 2.82 | 3.46 | 127 | NA | 3 | 0 |
| 4 | *Capra aegagrus hircus* | Vena | 80 | 37 | 43 | 0.9 | 0.4 | 0.8 | 4 | 5.0 | 52 | 3.4 | 2.33 | 2.69 | 136 | 4.7 | 0 | 0 |
| 5 | *Capra aegagrus hircus* | Leech | 95 | 46 | 49 | NA | 0.8 | 1.3 | 4 | 5.2 | 72 | 3.1 | 2.29 | 4.87 | 135 | 5.1 | 1 | 0 |
| 5 | *Capra aegagrus hircus* | Vena | 73 | 39 | 35 | NA | 0.7 | 1.1 | 4 | 5.8 | 60 | 3.6 | 2.04 | 3.47 | 136 | 5.6 | 0 | 0 |
| 6 | *Capra aegagrus hircus* | Leech | 81 | 46 | 35 | NA | 0.8 | 0.5 | 4 | 4.4 | 58 | 4.8 | 2.75 | 4.41 | 137 | 4.4 | 2 | 0 |
| 6 | *Capra aegagrus hircus* | Vena | 61 | 37 | 24 | NA | 0.6 | 0.5 | 4 | 4.8 | 57 | 5.0 | 2.29 | 3.40 | 143 | 4.7 | 1 | 0 |
| 7 | *Capra ibex* | Leech | 92 | 38 | 39 | 3.6 | 0.3 | 2.0 | NA | 4.6 | 65 | 1.2 | NA | 3.21 | 137 | 4.8 | 3 | 3 |
| 7 | *Capra ibex* | Vena | 66 | 32 | 33 | 1.9 | 0.3 | 2.1 | 5 | 7.2 | 91 | 7.1 | 2.09 | 3.94 | 142 | 5.2 | 0 | 0 |
| 8 | *Capra ibex* | Leech | 64 | 39 | 25 | 5.8 | 0.3 | 0.3 | 5 | 5.7 | 76 | 6.6 | 2.90 | 3.17 | 135 | 6.8 | 1 | 0 |
| 8 | *Capra ibex* | Vena | 52 | 33 | 20 | 4.2 | 0.3 | 0.3 | 5 | 6.3 | 82 | 6.8 | 2.54 | 2.49 | 140 | 6.6 | 0 | 0 |
| 9 | *Capra ibex* | Leech | 75 | 41 | 33 | 3.4 | 0.3 | 1.9 | 4 | 6.4 | 60 | 10.9 | 2.46 | 2.98 | 130 | 4.2 | 2 | 0 |
| 9 | *Capra ibex* | Vena | 70 | 39 | 31 | 2.6 | 0.3 | 1.9 | 6 | 6.8 | 84 | 11.9 | 2.26 | 3.23 | 138 | 5.0 | 1 | 0 |
| 10 | *Capra ibex* | Leech | 83 | 40 | 43 | 6.9 | 0.7 | 2.2 | NA | 4.1 | NA | 8.2 | 2.77 | 4.00 | 125 | NA | 3 | 0 |
| 10 | *Capra ibex* | Vena | 70 | 38 | 32 | 5.0 | 0.4 | 2.4 | 5 | 5.4 | 69 | 11 | 2.28 | 3.76 | 144 | 6.0 | 0 | 0 |
| 11 | *Equus caballus* | Leech | 88 | 37 | 51 | 2.6 | 0.3 | 0.1 | 16 | 4.1 | 140 | 4.6 | 3.47 | 0.93 | 128 | 4.6 | 0 | 0 |
| 11 | *Equus caballus* | Vena | 71 | 29 | 42 | 1.9 | 0.3 | 0.1 | 14 | 4.7 | 131 | 5.1 | 3.11 | 0.86 | 133 | 4.4 | 0 | 0 |
| 12 | *Equus caballus* | Leech | 116 | 48 | 67 | 3.3 | 0.6 | 0.2 | 16 | 2.6 | 106 | 9.8 | 3.81 | 2.13 | 127 | 4.1 | 0 | 0 |
| 12 | *Equus caballus* | Vena | 75 | 37 | 38 | 1.9 | 0.5 | 0.1 | 13 | 3.2 | 71 | 8.4 | 3.19 | 1.62 | 133 | 4.4 | 0 | 0 |
| 13 | *Equus caballus* | Leech | 112 | 48 | 64 | 4.1 | 0.3 | 0.3 | 20 | 3.3 | 156 | 4.3 | 3.79 | 2.34 | 134 | 4.6 | 0 | 0 |
| 13 | *Equus caballus* | Vena | 64 | 33 | 31 | 2.3 | 0.4 | 0.1 | 14 | 3.1 | 104 | 5.3 | 2.84 | 1.58 | 129 | 5.9 | 0 | 0 |
| 14 | *Equus quagga chapmani* | Leech | 72 | 50 | 22 | 2.9 | 0.5 | 0.1 | NA | 3.6 | 111 | 8.2 | 3.25 | 2.30 | 124 | NA | 3 | 0 |
| 14 | *Equus quagga chapmani* | Vena | 49 | 39 | 10 | 2.8 | 0.2 | 0.1 | 5 | 4.1 | 117 | 7.9 | 2.72 | 1.63 | 133 | 4.3 | 0 | 0 |
| 15 | *Oryctolagus cuniculus* | Leech | 63 | 21 | 42 | 1.7 | 0.7 | 3.1 | 4 | 5.7 | 108 | 5.1 | 3.58 | 2.56 | 139 | 4.3 | 1 | 0 |
| 15 | *Oryctolagus cuniculus* | Vena | 54 | 19 | 35 | 1.4 | 0.6 | 2.8 | 6 | 6.7 | 116 | 6.8 | 3.30 | 2.80 | 141 | 4.9 | 2 | 0 |
| 16 | *Oryctolagus cuniculus* | Leech | 63 | 21 | 42 | 1.7 | 0.7 | 3.1 | 4 | 5.7 | 108 | 5.1 | 3.58 | 2.56 | 139 | 4.3 | 1 | 0 |
| 16 | *Oryctolagus cuniculus* | Vena | 55 | 17 | 38 | 1.4 | 0.6 | 2.5 | 5 | 3.9 | 131 | 6.5 | 3.16 | 2.66 | 137 | 4.4 | 2 | 0 |
| 17 | *Oryctolagus cuniculus* | Leech | 69 | 24 | 44 | 2.2 | 1.0 | 3.7 | 5 | 3.8 | 103 | 2.5 | 3.49 | 3.63 | 133 | 3.3 | 2 | 0 |
| 17 | *Oryctolagus cuniculus* | Vena | 53 | 20 | 33 | 1.3 | 0.8 | 3.1 | 4 | 4.4 | 78 | 5.1 | 3.09 | 3.27 | 139 | 3.9 | 1 | 0 |
| 18 | *Oryctolagus cuniculus* | Leech | 57 | 21 | 35 | 2.4 | 0.7 | 2.3 | 5 | 4.4 | 97 | 7 | 3.75 | 2.46 | 132 | 3.4 | 0 | 0 |
| 18 | *Oryctolagus cuniculus* | Vena | 49 | 20 | 29 | 1.9 | 0.6 | 2.2 | 4 | 5.1 | 129 | 9.6 | 3.18 | 2.57 | 139 | 4.2 | 1 | 0 |
| 19 | *Oryctolagus cuniculus* | Leech | 63 | 25 | 39 | 2.6 | 1.2 | 2.8 | 5 | 4.9 | 76 | 9.2 | 3.18 | 2.85 | 132 | NA | 3 | 0 |
| 19 | *Oryctolagus cuniculus* | Vena | 49 | 21 | 28 | 2.2 | 1.0 | 2.4 | 4 | 5.1 | 97 | 9.7 | 2.96 | 2.67 | 135 | 4.7 | 2 | 0 |
| 20 | *Oryctolagus cuniculus* | Leech | 77 | 29 | 48 | 3.4 | 1.0 | 4.1 | 3 | 4.4 | 122 | 7.7 | 3.57 | 3.52 | 135 | 3.2 | 1 | 0 |
| 20 | *Oryctolagus cuniculus* | Vena | 50 | 21 | 29 | 2.0 | 0.8 | 3.0 | 3 | 4.7 | 82 | 9.1 | 3.14 | 2.66 | 141 | 3.5 | 0 | 0 |
| 21 | *Oryctolagus cuniculus* | Leech | 63 | 25 | 37 | 2.8 | 1.1 | 2.8 | 3 | 4.5 | 106 | 7.1 | 3.41 | 2.94 | 133 | 3.6 | 0 | 0 |
| 21 | *Oryctolagus cuniculus* | Vena | 48 | 21 | 26 | 1.7 | 0.9 | 2.4 | 3 | 4.7 | 96 | 7.5 | 3.14 | 2.50 | 139 | 4.0 | 1 | 0 |
| 22 | *Ovis aries* | Leech | 111 | 51 | 60 | 7.2 | 0.8 | 0.2 | 4 | 4.9 | 53 | 0.3 | 2.90 | 2.75 | 132 | 4.7 | 1 | 0 |
| 22 | *Ovis aries* | Vena | 65 | 34 | 31 | 4.6 | 0.5 | 0.1 | 4 | 5.3 | 41 | 2.9 | 2.26 | 1.60 | 134 | 4.9 | 0 | 0 |
| 23 | *Ovis aries* | Leech | 117 | 46 | 71 | 8.1 | 0.9 | 0.2 | 4 | 4.2 | 31 | 0.8 | 2.89 | 3.02 | 125 | 5.1 | 1 | 0 |
| 23 | *Ovis aries* | Vena | 72 | 34 | 39 | 3.5 | 0.6 | 0.1 | 3 | 4.5 | 18 | 3.1 | 2.43 | 2.23 | 129 | 6.8 | 3 | 0 |
| 24 | *Ovis aries* | Leech | 87 | 40 | 47 | 5.9 | 0.6 | 0.4 | 7 | 5.2 | 104 | 3.6 | 2.66 | 2.74 | 137 | 4.2 | 0 | 0 |
| 24 | *Ovis aries* | Vena | 72 | 35 | 37 | NA | 0.5 | 0.4 | 5 | 5.0 | 73 | 4.5 | 2.49 | 2.02 | 138 | 4.5 | 0 | 0 |
| 25 | *Ovis aries* | Leech | 96 | 51 | 45 | 6.4 | 0.8 | 0.1 | 4 | 4.4 | 68 | 1.2 | 3.06 | 2.43 | 131 | 5.3 | 2 | 0 |
| 25 | *Ovis aries* | Vena | 66 | 38 | 28 | 4.3 | 0.6 | 0.2 | 4 | 4.7 | 41 | 3.6 | 2.57 | 1.75 | 136 | 4.8 | 0 | 0 |
| 26 | *Ovis aries* | Leech | 81 | 43 | 39 | 5.5 | 0.7 | 0.2 | 5 | 2.7 | 85 | 2.5 | 2.33 | 3.76 | 134 | 5.1 | 0 | 0 |
| 26 | *Ovis aries* | Vena | 59 | 32 | 27 | 4.4 | 0.5 | 0.1 | 6 | 2.9 | 83 | 3.7 | 1.94 | 3.08 | 137 | 5.0 | 1 | 0 |
| 27 | *Ovis aries* | Leech | 82 | 43 | 39 | 2.1 | 0.6 | 0.1 | 4 | 4.2 | 67 | 2.8 | 2.65 | 2.74 | 136 | 4.0 | 2 | 0 |
| 27 | *Ovis aries* | Vena | 67 | 36 | 31 | 1.8 | 0.5 | 0.1 | 5 | 4.6 | 61 | 3.9 | 2.42 | 1.94 | 141 | 4.2 | 0 | 0 |
| 28 | *Ovis aries* | Leech | 97 | 47 | 50 | 4.6 | 0.9 | 0.2 | 4 | 3.1 | 73 | 3.4 | 2.95 | 3.14 | 133 | 5.5 | 2 | 0 |
| 28 | *Ovis aries* | Vena | 67 | 36 | 31 | 3.5 | 0.7 | 0.2 | 4 | 3.2 | 79 | 5.1 | 2.38 | 2.26 | 141 | 5.3 | 0 | 0 |
| 29 | *Ovis aries* | Leech | 114 | 56 | 57 | 6.7 | 1.1 | 0.3 | 4 | 3.3 | 94 | 0.3 | 2.96 | 4.37 | 137 | 4.9 | 3 | 0 |
| 29 | *Ovis aries* | Vena | 64 | 36 | 27 | 4.0 | 0.7 | 0.2 | 4 | 3.4 | 60 | 3.8 | 2.25 | 2.32 | 140 | 4.3 | 0 | 0 |
| 30 | *Ovis aries* | Leech | 67 | 41 | 26 | 6.2 | 0.3 | 0.3 | 5 | 5.8 | 61 | 6.9 | 2.95 | 3.28 | 141 | 6.7 | 1 | 0 |
| 30 | *Ovis aries* | Vena | 49 | 33 | 16 | 4.3 | 0.2 | 0.2 | 5 | 6.3 | 63 | 6.6 | 2.52 | 2.47 | 142 | 6.4 | 0 | 0 |
| 31 | *Rattus norvegicus* | Leech | 76 | 57 | 18 | 5.5 | 2.4 | 11.5 | 5 | 4.9 | 61 | 11.2 | 2.89 | 2.99 | 128 | 8.1 | 2 | 0 |
| 31 | *Rattus norvegicus* | Vena | 59 | 46 | 13 | 3.7 | 0.9 | 9.4 | 4 | 5.7 | 36 | 12.5 | 2.75 | 2.85 | 129 | 6.6 | 3 | 0 |
| 32 | *Vicugna pacos* | Leech | 90 | 52 | 38 | 1.7 | 0.6 | 12.4 | 4 | 5.9 | 186 | 5.4 | 2.37 | 2.59 | 139 | 4.5 | 0 | 0 |
| 32 | *Vicugna pacos* | Vena | 62 | 39 | 23 | 0.6 | 0.6 | 9.4 | 4 | 6.4 | 137 | 6.6 | 2.02 | 2.02 | 145 | 4.9 | 0 | 0 |
| 33 | *Vicugna pacos* | Leech | 93 | 55 | 38 | 3.0 | 0.3 | 14.2 | 4 | 5.6 | 155 | 4.3 | 2.50 | 1.93 | 134 | 4.6 | 2 | 0 |
| 33 | *Vicugna pacos* | Vena | 69 | 46 | 24 | 1.6 | 0.3 | 12.5 | 4 | 6.1 | 143 | 7.1 | 2.29 | 1.82 | 147 | 5.5 | 1 | 0 |
| 34 | *Vicugna pacos* | Leech | 74 | 48 | 26 | 0.7 | 0.5 | 15.7 | 4 | 6.6 | 167 | 7.2 | 2.39 | 1.96 | 141 | 4.5 | 0 | 0 |
| 34 | *Vicugna pacos* | Vena | 64 | 43 | 21 | 0.5 | 0.5 | 14.5 | 5 | 7.0 | 156 | 6.3 | 2.39 | 1.90 | 146 | 5.1 | 0 | 0 |
| 35 | *Vicugna pacos* | Leech | 72 | 51 | 21 | 2.3 | 0.7 | 10.0 | 4 | 5.1 | 191 | 7.3 | 2.47 | 2.18 | 139 | 4.9 | 0 | 0 |
| 35 | *Vicugna pacos* | Vena | 60 | 46 | 15 | 1.8 | 0.6 | 9.2 | 3 | 5.3 | 156 | 6.8 | 2.29 | 1.76 | 143 | 4.8 | 0 | 0 |
| 36 | *Vicugna pacos* | Leech | 75 | 52 | 23 | 1.4 | 0.3 | 12.1 | 4 | 5.6 | 148 | 5.40 | 2.78 | 3.20 | 146 | NA | 0 | 0 |
| 36 | *Vicugna pacos* | Vena | 66 | 51 | 15 | 0.5 | 0.2 | 9.6 | 4 | 5 | 115 | 6.4 | 2.49 | 2.25 | 142 | 4.8 | 2 | 0 |
| 37 | *Vicugna pacos* | Leech | 89 | 65 | NA | 2.0 | 0.7 | 21.4 | 3 | 4.2 | 262 | 8.8 | 3.09 | 2.82 | 142 | 4.4 | 0 | 0 |
| 37 | *Vicugna pacos* | Vena | 56 | 49 | 7 | 1.1 | 0.4 | 14.1 | 3 | 5.5 | 161 | 9.4 | 2.51 | 2.00 | 149 | 5.0 | 0 | 0 |
| 38 | *Vicugna pacos* | Leech | 72 | 53 | 19 | 1.7 | 0.3 | 11.1 | 2 | 4.5 | 179 | 6.8 | 2.42 | 1.48 | 146 | 4.1 | 0 | 0 |
| 38 | *Vicugna pacos* | Vena | 64 | 51 | 13 | 1.4 | 0.3 | 10.4 | 3 | 4.6 | 160 | 7.2 | 2.21 | 1.50 | 148 | 4.4 | 0 | 0 |
| 39 | *Oryctolagus cuniculus* | Leech | 86 | 23 | 63 | 1.4 | 0.9 | 4.1 | NA | 5.7 | 108 | 3.5 | 3.44 | 2.09 | 137 | NA | 3 | 0 |
| 39 | *Oryctolagus cuniculus* | Vena | 69 | 18 | 51 | 1.0 | 0.7 | 3.5 | 3 | 6.4 | 113 | 6.2 | 3.23 | 1.95 | 146 | 2.9 | 0 | 0 |
| 40 | *Oryctolagus cuniculus* | Leech | 55 | 20 | 35 | 3.7 | 1.0 | 5.2 | 1 | 5 | 108 | 7.5 | 3.27 | 2.60 | 138 | 2.8 | 0 | 0 |
| 40 | *Oryctolagus cuniculus* | Vena | 42 | 17 | 26 | 3.0 | 0.8 | 4.5 | 2 | 5.4 | 146 | 8 | 3.18 | 2.63 | 143 | 3.3 | 0 | 0 |
| 41 | *Oryctolagus cuniculus* | Leech | 73 | 26 | 47 | 3.8 | 0.7 | 3.1 | 2 | 5.2 | 73 | 5.3 | 3.83 | 2.86 | 140 | NA | 3 | 0 |
| 41 | *Oryctolagus cuniculus* | Vena | 53 | 21 | 32 | 2.7 | 0.5 | 2.7 | 2 | 5.6 | 92 | 7.2 | 3.26 | 2.47 | 143 | 3.2 | 0 | 0 |
| 42 | *Oryctolagus cuniculus* | Leech | 67 | 20 | 47 | 2.6 | 0.7 | 3.3 | 4 | 4 | 86 | 6.9 | 3.32 | 2.22 | 132 | 3.3 | 1 | 0 |
| 42 | *Oryctolagus cuniculus* | Vena | 56 | 18 | 38 | 1.6 | 0.6 | 2.9 | 4 | 4.9 | 99 | 8.6 | 3.32 | 2.35 | 141 | 3.9 | 0 | 0 |
| 43 | *Oryctolagus cuniculus* | Leech | 75 | 27 | 48 | 3.5 | 1.0 | 3.2 | 3 | 5 | 154 | 8 | 3.67 | 3.13 | 132 | 3.3 | 1 | 0 |
| 43 | *Oryctolagus cuniculus* | Vena | 52 | 19 | 32 | 2.2 | 0.6 | 1.8 | 4 | 4.9 | 89 | 8.4 | 3.22 | 2.23 | 140 | 4.1 | 3 | 0 |
| 44 | *Oryctolagus cuniculus* | Leech | 75 | 25 | 50 | 4.1 | 0.8 | 3.7 | 2 | 5.5 | 97 | 6.9 | 3.26 | 3.08 | 138 | 3.5 | 1 | 0 |
| 44 | *Oryctolagus cuniculus* | Vena | 51 | 20 | 31 | 4.7 | 0.7 | 2.8 | 2 | 6.6 | 71 | 9.4 | 2.86 | 2.73 | 144 | 3.6 | 0 | 0 |
| 45 | *Oryctolagus cuniculus* | Leech | 80 | 26 | 54 | 2.5 | 1.1 | 2.9 | 2 | 5.9 | 95 | 6.3 | 3.31 | 2.84 | 139 | 3.7 | 0 | 0 |
| 45 | *Oryctolagus cuniculus* | Vena | 62 | 22 | 40 | 1.9 | 1.0 | 2.4 | 1 | 6.9 | 82 | 8.6 | 3.00 | 2.55 | 143 | 4.6 | 2 | 0 |
| 46 | *Macropus rufogriseus* | Leech | 68 | 35 | 32 | 7.2 | 2.5 | 7.2 | 30 | 2.7 | 64 | 3.5 | 2.33 | 1.63 | 124 | 3.8 | 2 | 0 |
| 46 | *Macropus rufogriseus* | Vena | 51 | 29 | 22 | 5.8 | 2.1 | 6.2 | 28 | 4 | 67 | 4.7 | 2.16 | 1.40 | 139 | 3.8 | 0 | 0 |
| 47 | *Dolichotis patagonum* | Leech | 64 | 32 | 32 | 2.5 | 0.4 | 28.2 | 6 | 3.5 | 70 | 11.3 | 3.10 | 1.68 | 125 | 5.6 | 2 | 0 |
| 47 | *Dolichotis patagonum* | Vena | 52 | 32 | 20 | 1.2 | 0.5 | 26.9 | 5 | 4.1 | 80 | 11.8 | 2.83 | 1.46 | 146 | 6.1 | 1 | 0 |
| 48 | *Oryctolagus cuniculus* | Leech | 60 | 23 | 38 | 1.9 | 0.5 | 2.8 | 3 | 6 | 96 | 6.6 | 3.31 | 2.56 | 134 | 4.0 | 0 | 0 |
| 48 | *Oryctolagus cuniculus* | Vena | 57 | 23 | 35 | 1.9 | 0.5 | 2.8 | 2 | 7.1 | 91 | 7.8 | 3.28 | 2.63 | 142 | 4.6 | 0 | 0 |
| 49 | *Oryctolagus cuniculus* | Leech | 66 | 19 | 47 | 1.4 | 0.6 | 2.6 | 3 | 5.4 | 102 | 5.2 | 3.23 | 2.27 | 130 | 3.9 | 0 | 0 |
| 49 | *Oryctolagus cuniculus* | Vena | 60 | 18 | 42 | 1.3 | 0.1 | 2.4 | 4 | 5.9 | 108 | 7.1 | 3.25 | 2.30 | 138 | 4.0 | 0 | 0 |
| 50 | *Oryctolagus cuniculus* | Leech | 53 | 21 | 33 | 1.9 | 0.7 | 2.6 | 3 | 5.1 | 82 | 7.4 | 3.25 | 2.71 | 141 | 5.2 | 1 | 0 |
| 50 | *Oryctolagus cuniculus* | Vena | 56 | 20 | 36 | 2.2 | 0.7 | 2.5 | 3 | 4.4 | 72 | 6.5 | 3.16 | 2.23 | 130 | 4.5 | 0 | 0 |
| 51 | *Oryctolagus cuniculus* | Leech | 69 | 26 | 43 | 3.1 | 1.0 | 3.5 | 3 | 5.2 | 79 | 5.8 | 3.88 | 2.63 | 129 | 3.8 | 2 | 0 |
| 51 | *Oryctolagus cuniculus* | Vena | 52 | 22 | 30 | 2.7 | 0.8 | 3.0 | 3 | 6.1 | 59 | 7.9 | 3.55 | 2.70 | 142 | 4.2 | 0 | 0 |
| 52 | *Oryctolagus cuniculus* | Leech | 70 | 25 | 45 | 3.6 | 1.0 | 3.0 | 3 | 6.3 | 96 | 7.1 | 3.42 | 3.08 | 133 | 3.5 | 1 | 0 |
| 52 | *Oryctolagus cuniculus* | Vena | 55 | 22 | 33 | 2.9 | 0.7 | 2.7 | 3 | 7.3 | 97 | 9.4 | 3.04 | 2.88 | 143 | 5.0 | 0 | 0 |
| 53 | *Oryctolagus cuniculus* | Leech | 60 | 23 | 37 | 3.7 | 0.8 | 2.5 | 2 | 4.1 | 71 | 6.5 | 3.37 | 3.09 | 128 | 3.4 | 0 | 0 |
| 53 | *Oryctolagus cuniculus* | Vena | 52 | 22 | 30 | 3.2 | 0.8 | 2.2 | 2 | 4.9 | 88 | 9.5 | 3.16 | 3.09 | 143 | 4.1 | 1 | 0 |
| 54 | *Oryctolagus cuniculus* | Leech | 65 | 23 | 42 | 2.6 | 1.1 | 2.9 | 2 | 3.8 | 102 | 5.9 | 3.50 | 3.39 | 129 | 3.5 | 0 | 0 |
| 54 | *Oryctolagus cuniculus* | Vena | 51 | 21 | 30 | 1.8 | 1.0 | 2.4 | 2 | 5.3 | 103 | 10.3 | 3.13 | 3.17 | 144 | 4.0 | 0 | 0 |
| 55 | *Oryctolagus cuniculus* | Leech | 56 | 23 | 33 | 3.4 | 1.1 | 2.5 | 2 | 5.6 | 126 | 6.9 | 3.11 | 3.50 | 136 | 4.1 | 0 | 0 |
| 55 | *Oryctolagus cuniculus* | Vena | 47 | 21 | 26 | 3.1 | 1.0 | 2.2 | 2 | 6.3 | 89 | 9.1 | 2.81 | 3.19 | 144 | 4.9 | 0 | 0 |
| 56 | *Oryctolagus cuniculus* | Leech | 61 | 24 | 38 | 4.0 | 0.9 | 3.1 | 2 | 5 | 122 | 5.4 | 3.49 | 3.10 | 130 | 4.0 | 1 | 0 |
| 56 | *Oryctolagus cuniculus* | Vena | 50 | 21 | 28 | 3.4 | 0.7 | 2.6 | 2 | 6.2 | 124 | 8.5 | 3.16 | 3.00 | 144 | 4.9 | 1 | 0 |
| 57 | *Oryctolagus cuniculus* | Leech | 65 | 26 | 39 | 2.3 | 0.7 | 4.3 | 2 | 6.6 | 120 | 7.1 | 3.43 | 2.78 | 137 | 3.5 | 0 | 0 |
| 57 | *Oryctolagus cuniculus* | Vena | 56 | 25 | 32 | 2.1 | 0.7 | 4.0 | 2 | 7.5 | 113 | 10.5 | 3.12 | 2.78 | 146 | 4.5 | 0 | 0 |
| 58 | *Oryctolagus cuniculus* | Leech | 74 | 25 | 48 | 2.2 | 0.8 | 2.9 | 2 | 4.4 | 72 | 3 | 3.43 | 2.68 | 119 | 4.1 | 1 | 0 |
| 58 | *Oryctolagus cuniculus* | Vena | 61 | 24 | 37 | 1.7 | 0.7 | 2.5 | 2 | 5.6 | 65 | 6.8 | 3.16 | 2.59 | 137 | 2.9 | 0 | 0 |
| 59 | *Oryctolagus cuniculus* | Leech | 61 | 27 | 34 | 2.7 | 0.5 | 3.5 | 3 | 5.4 | 80 | 5.4 | 3.41 | 2.65 | 135 | 3.4 | 2 | 0 |
| 59 | *Oryctolagus cuniculus* | Vena | 55 | 24 | 31 | 2.3 | 0.8 | 3.0 | 2 | 6.1 | 76 | 6.8 | 3.30 | 2.68 | 138 | 4.4 | 2 | 0 |
| 60 | *Oryctolagus cuniculus* | Leech | 70 | 26 | 43 | 2.8 | 0.7 | 3.5 | 4 | 3.9 | 73 | 4.5 | 3.86 | 2.85 | 133 | 4.6 | 2 | 0 |
| 60 | *Oryctolagus cuniculus* | Vena | 49 | 21 | 27 | 2.1 | 0.5 | 2.9 | 3 | 5.1 | 82 | 10.6 | 3.31 | 2.76 | 146 | 4.7 | 0 | 0 |
| 61 | *Oryctolagus cuniculus* | Leech | 62 | 21 | 42 | 1.6 | 0.5 | 3.1 | 5 | 4.5 | 77 | 7.8 | 3.07 | 2.71 | 131 | 3.7 | 0 | 0 |
| 61 | *Oryctolagus cuniculus* | Vena | 53 | 20 | 33 | 1.3 | 0.3 | 3.0 | 3 | 5.8 | 102 | 12.5 | 2.80 | 2.99 | 146 | 4.7 | 0 | 0 |
| 62 | *Oryctolagus cuniculus* | Leech | 86 | 23 | 62 | 1.6 | 1.0 | 2.7 | 4 | 6.1 | 97 | 5.2 | 3.34 | 2.67 | 141 | 3.5 | 2 | 0 |
| 62 | *Oryctolagus cuniculus* | Vena | 59 | 21 | 28 | 1.0 | 0.7 | 2.2 | 4 | 8.3 | 103 | 8.8 | 2.87 | 2.75 | 142 | 3.9 | 2 | 0 |
| 63 | *Sus scrofa* | Leech | 75 | 43 | 32 | 2.5 | 1.2 | 12.1 | 14 | 3.8 | 83 | 7.4 | 2.82 | 2.21 | 133 | 4.0 | 1 | 0 |
| 63 | *Sus scrofa* | Vena | 72 | 43 | 29 | NA | 1.2 | 11.7 | 14 | NA | NA | 6.9 | 2.63 | 1.90 | 134 | 4.5 | 0 | 0 |

**Supplementary Table 2** Results of 13 haematology parameters in individual zoo animals (n = 63) in blood samples collected by leeches and venipuncture. White blood cell count (WBC, 10^9^/L), lymphocyte count (LYM, 10^9^/L; proportion as LYM%), monocyte count (MON, 10^9^/L; proportion as MON%), neutrophil count (NEU, 10^9^/L; proportion as NEU%), red blood cell count (RBC, 10^12^/L), haemoglobin (HGB, g/dl), haematocrit (HCT, %), mean cell volume (MCV, fL), mean corpuscular haemoglobin (MCH, pg), and mean corpuscular haemoglobin concentration (MCHC, g/dL), not available (NA), leech sample (Leech), venipuncture sample (Vena).

| No. | Species | Method | WBC | LYM | MON | NEU | LYM% | MON% | NEU% | RBC | HGB | HCT | MCV | MCH | MCHC |
| --- | --- | --- | --- | --- | --- | --- | --- | --- | --- | --- | --- | --- | --- | --- | --- |
| 1 | *Capra aegagrus hircus* | Leech | 9.64 | 4.94 | 0.10 | 4.60 | 51.3 | 1.04 | 47.7 | 14.90 | 10.5 | 26.9 | 18 | 7.0 | 39.0 |
| 1 | *Capra aegagrus hircus* | Vena | 11.74 | 5.53 | 0.07 | 6.14 | 47.1 | 0.60 | 52.3 | 13.73 | 9.5 | 24.7 | 18 | 6.9 | 38.4 |
| 2 | *Capra aegagrus hircus* | Leech | 14.97 | 8.24 | 0.11 | 6.62 | 55.1 | 0.73 | 44.2 | 15.16 | 9.9 | 25.8 | 17 | 6.6 | 38.6 |
| 2 | *Capra aegagrus hircus* | Vena | 11.81 | 5.70 | 0.10 | 6.00 | 48.3 | 0.85 | 50.9 | 16.04 | 10.7 | 27.3 | 17 | 6.7 | 39.0 |
| 3 | *Capra aegagrus hircus* | Leech | 4.66 | 2.92 | 0.03 | 1.71 | 62.7 | 0.64 | 36.8 | NA | NA | NA | NA | NA | NA |
| 3 | *Capra aegagrus hircus* | Vena | 4.73 | 2.09 | 0.04 | 2.59 | 44.2 | 0.85 | 54.9 | 13.87 | 8.8 | 23.4 | 17 | 6.3 | 37.5 |
| 4 | *Capra aegagrus hircus* | Leech | 11.33 | 4.48 | 0.06 | 6.79 | 39.5 | 0.53 | 59.9 | 13.64 | 10.3 | 26.4 | 19 | 7.6 | 39.2 |
| 4 | *Capra aegagrus hircus* | Vena | 10.98 | 4.45 | 0.07 | 6.47 | 40.5 | 0.64 | 58.9 | 12.08 | 8.9 | 23.4 | 19 | 7.4 | 38.0 |
| 5 | *Capra aegagrus hircus* | Leech | 11.51 | 6.83 | 0.09 | 4.59 | 59.3 | 0.78 | 39.9 | 16.15 | 10.1 | 28.5 | 18 | 6.3 | 35.6 |
| 5 | *Capra aegagrus hircus* | Vena | 9.79 | 5.00 | 0.09 | 4.71 | 51.0 | 0.92 | 48.1 | 14.71 | 9.3 | 25.5 | 17 | 6.3 | 36.4 |
| 6 | *Capra aegagrus hircus* | Leech | 25.49 | 14.18 | 0.22 | 11.09 | 55.6 | 0.86 | 43.5 | 18.43 | 12.3 | 28.9 | 16 | 6.7 | 42.7 |
| 6 | *Capra aegagrus hircus* | Vena | 19.60 | 9.45 | 0.18 | 9.96 | 48.2 | 0.92 | 50.8 | 16.52 | 11.1 | 25.1 | 15 | 6.7 | 44.1 |
| 7 | *Capra ibex* | Leech | 5.16 | 1.43 | 0.04 | 3.69 | 27.7 | 0.78 | 71.5 | NA | NA | NA | 20 | 6.3 | 31.4 |
| 7 | *Capra ibex* | Vena | 3.73 | 2.17 | 0.02 | 1.54 | 58.1 | 0.54 | 41.4 | 19.86 | 14.7 | 42.7 | 22 | 7.4 | 34.5 |
| 8 | *Capra ibex* | Leech | 10.58 | 3.10 | 0.09 | 7.39 | 29.3 | 0.85 | 69.8 | 17.29 | 16.7 | 42.6 | 25 | 9.6 | 39.1 |
| 8 | *Capra ibex* | Vena | 12.96 | 0.50 | 0.08 | 12.37 | 3.9 | 0.62 | 95.5 | 15.61 | 14.5 | 38.9 | 25 | 9.3 | 37.4 |
| 9 | *Capra ibex* | Leech | 15.48 | 10.90 | 0.08 | 14.32 | 7.0 | 0.52 | 92.5 | 16.28 | 13.6 | 35.1 | 22 | 8.4 | 38.8 |
| 9 | *Capra ibex* | Vena | 16.91 | 1.95 | 0.10 | 14.86 | 11.5 | 0.59 | 87.9 | 16.49 | 14.1 | 35.4 | 21 | 8.5 | 38.8 |
| 10 | *Capra ibex* | Leech | 18.72 | 9.36 | 0.14 | 9.21 | 50.0 | 0.75 | 49.2 | 16.32 | 13.2 | 32.8 | 20 | 8.1 | 40.2 |
| 10 | *Capra ibex* | Vena | 16.26 | 4.47 | 0.10 | 11.70 | 27.5 | 0.62 | 71.9 | 17.18 | 12.8 | 34.7 | 20 | 7.5 | 37.0 |
| 11 | *Equus caballus* | Leech | 9.25 | 4.03 | 0.05 | 4.92 | 43.6 | 0.54 | 53.2 | 5.89 | 10.1 | 27.5 | 47 | 17.2 | 36.8 |
| 11 | *Equus caballus* | Vena | 6.79 | 2.27 | 0.31 | 4.05 | 33.4 | 4.57 | 59.7 | 5.91 | 10.4 | 27.6 | 47 | 17.6 | 37.7 |
| 12 | *Equus caballus* | Leech | 9.50 | 3.76 | 0.36 | 5.34 | 39.5 | 3.79 | 56.2 | 8.77 | 15.6 | 43.3 | 49 | 17.8 | 35.9 |
| 12 | *Equus caballus* | Vena | 7.80 | 2.71 | 0.30 | 4.77 | 34.7 | 3.85 | 61.2 | 7.97 | 13.6 | 38.1 | 48 | 17.0 | 35.6 |
| 13 | *Equus caballus* | Leech | 11.28 | 5.88 | 0.06 | 5.09 | 52.1 | 0.53 | 45.2 | 8.92 | 15.0 | 38.5 | 43 | 16.8 | 38.9 |
| 13 | *Equus caballus* | Vena | 6.71 | 3.05 | 0.30 | 3.09 | 45.4 | 4.47 | 46.0 | 7.31 | 11.9 | 31.5 | 43 | 16.3 | 37.8 |
| 14 | *Equus quagga chapmani* | Leech | 11.86 | 9.35 | 0.42 | 1.81 | 78.8 | 3.54 | 15.3 | 9.62 | 13.3 | 37.3 | 39 | 13.8 | 35.5 |
| 14 | *Equus quagga chapmani* | Vena | 9.00 | 7.30 | 0.08 | 1.48 | 81.2 | 0.89 | 16.5 | 8.18 | 11.0 | 32.6 | 40 | 13.4 | 33.7 |
| 15 | *Oryctolagus cuniculus* | Leech | 13.84 | 6.11 | 0.17 | 7.57 | 44.1 | 1.23 | 54.7 | 6.47 | 12.1 | 37.6 | 58 | 18.7 | 32.1 |
| 15 | *Oryctolagus cuniculus* | Vena | 6.26 | 3.28 | 0.12 | 2.86 | 52.4 | 1.92 | 45.6 | 4.95 | 9.1 | 29.5 | 60 | 18.3 | 30.7 |
| 16 | *Oryctolagus cuniculus* | Leech | 16.72 | 2.33 | 1.85 | 12.54 | 13.9 | 11.06 | 75.0 | 6.27 | 11.8 | 37.0 | 59 | 18.8 | 31.9 |
| 16 | *Oryctolagus cuniculus* | Vena | 14.94 | 3.46 | 0.86 | 10.62 | 23.2 | 5.76 | 71.1 | 5.45 | 10.4 | 32.8 | 60 | 19.1 | 31.8 |
| 17 | *Oryctolagus cuniculus* | Leech | 7.90 | 2.19 | 0.83 | 4.88 | 27.7 | 10.51 | 61.8 | 6.79 | 13.7 | 40.0 | 59 | 20.2 | 34.3 |
| 17 | *Oryctolagus cuniculus* | Vena | 5.90 | 1.04 | 0.44 | 4.42 | 17.6 | 7.46 | 74.9 | 6.00 | 10.9 | 35.2 | 59 | 18.2 | 31.3 |
| 18 | *Oryctolagus cuniculus* | Leech | 10.87 | 3.51 | 0.89 | 6.47 | 23.3 | 8.19 | 59.5 | 6.21 | 11.7 | 38.3 | 62 | 18.9 | 30.6 |
| 18 | *Oryctolagus cuniculus* | Vena | 2.01 | 0.84 | 0.16 | 1.00 | 42.0 | 7.96 | 50.1 | 3.99 | 6.9 | 25.4 | 64 | 17.2 | 27.1 |
| 19 | *Oryctolagus cuniculus* | Leech | 12.09 | 3.98 | 0.09 | 8.02 | 32.9 | 0.74 | 66.4 | 6.38 | 13.7 | 37.9 | 59 | 21.4 | 36.0 |
| 19 | *Oryctolagus cuniculus* | Vena | 5.24 | 1.49 | 0.43 | 3.33 | 28.4 | 8.21 | 63.4 | 5.95 | 10.8 | 34.8 | 59 | 18.1 | 31.0 |
| 20 | *Oryctolagus cuniculus* | Leech | 9.92 | 4.63 | 1.02 | 4.27 | 46.6 | 10.28 | 43.1 | 7.46 | 14.8 | 43.3 | 58 | 19.8 | 34.1 |
| 20 | *Oryctolagus cuniculus* | Vena | 7.45 | 2.17 | 0.39 | 4.89 | 29.1 | 5.23 | 65.7 | 5.37 | 9.8 | 31.2 | 58 | 18.3 | 31.6 |
| 21 | *Oryctolagus cuniculus* | Leech | 17.40 | 7.34 | 0.13 | 9.93 | 42.2 | 0.75 | 57.1 | 6.33 | 13.2 | 37.8 | 60 | 20.9 | 34.9 |
| 21 | *Oryctolagus cuniculus* | Vena | 8.10 | 1.10 | 0.53 | 6.47 | 13.6 | 6.54 | 79.9 | 5.83 | 11.2 | 34.6 | 59 | 19.2 | 32.3 |
| 22 | *Ovis aries* | Leech | 6.75 | 4.40 | 0.03 | 2.31 | 65.2 | 0.44 | 34.3 | 8.94 | 10.8 | 26.2 | 29 | 12.1 | 41.2 |
| 22 | *Ovis aries* | Vena | 3.94 | 2.57 | 0.02 | 1.35 | 65.2 | 0.51 | 34.3 | 8.27 | 9.6 | 24.0 | 29 | 11.6 | 40.0 |
| 23 | *Ovis aries* | Leech | 9.30 | 5.53 | 0.05 | 3.73 | 59.4 | 0.54 | 40.1 | 10.36 | 12.4 | 29.6 | 29 | 11.9 | 41.8 |
| 23 | *Ovis aries* | Vena | 5.34 | 2.71 | 0.03 | 2.60 | 50.7 | 0.56 | 48.8 | 7.46 | 8.6 | 19.8 | 27 | 11.6 | 43.7 |
| 24 | *Ovis aries* | Leech | 9.29 | 4.44 | 0.05 | 4.80 | 47.8 | 0.54 | 51.7 | 9.55 | 11.8 | 27.9 | 29 | 12.4 | 42.4 |
| 24 | *Ovis aries* | Vena | 8.22 | 3.69 | 0.04 | 4.49 | 44.9 | 0.49 | 54.6 | 9.21 | 11.4 | 27.0 | 29 | 12.3 | 42.1 |
| 25 | *Ovis aries* | Leech | 6.64 | 5.49 | 0.03 | 1.12 | 82.7 | 0.45 | 16.8 | 9.66 | 12.3 | 30.0 | 31 | 12.8 | 41.1 |
| 25 | *Ovis aries* | Vena | 4.56 | 3.51 | 0.02 | 1.03 | 76.9 | 0.44 | 22.6 | 7.99 | 10.0 | 24.3 | 30 | 12.5 | 41.2 |
| 26 | *Ovis aries* | Leech | 5.90 | 2.99 | 0.03 | 2.88 | 50.7 | 0.51 | 48.8 | 9.08 | 12.8 | 29.1 | 32 | 14.1 | 44.0 |
| 26 | *Ovis aries* | Vena | 4.01 | 1.91 | 0.02 | 2.07 | 47.8 | 0.50 | 51.7 | 7.95 | 10.7 | 25.5 | 32 | 13.4 | 41.8 |
| 27 | *Ovis aries* | Leech | 7.36 | 5.44 | 0.04 | 1.88 | 74.0 | 0.54 | 25.6 | 10.01 | 13.5 | 29.8 | 30 | 13.4 | 45.2 |
| 27 | *Ovis aries* | Vena | 6.70 | 4.96 | 0.03 | 1.71 | 74.0 | 0.45 | 25.6 | 9.38 | 11.8 | 27.8 | 30 | 12.5 | 42.4 |
| 28 | *Ovis aries* | Leech | 13.22 | 7.86 | 0.07 | 5.30 | 59.4 | 0.53 | 40.1 | 10.31 | 13.4 | 31.9 | 31 | 13.0 | 42.1 |
| 28 | *Ovis aries* | Vena | 6.78 | 4.82 | 0.03 | 1.93 | 71.0 | 0.44 | 28.5 | 8.55 | 11.6 | 26.1 | 31 | 13.6 | 44.5 |
| 29 | *Ovis aries* | Leech | 22.18 | 13.18 | 0.11 | 8.89 | 59.4 | 0.50 | 40.1 | 11.90 | 15.7 | 35.8 | 30 | 13.2 | 44.0 |
| 29 | *Ovis aries* | Vena | 8.06 | 5.96 | 0.04 | 2.06 | 74.0 | 0.50 | 25.6 | 8.77 | 10.2 | 24.8 | 28 | 11.7 | 41.2 |
| 30 | *Ovis aries* | Leech | 11.94 | 9.87 | 0.06 | 2.01 | 82.7 | 0.50 | 16.8 | 9.70 | 10.2 | 25.8 | 27 | 10.5 | 39.4 |
| 30 | *Ovis aries* | Vena | NA | NA | NA | NA | NA | NA | NA | 8.75 | 10.0 | 22.6 | 26 | 11.4 | 44.1 |
| 31 | *Rattus norvegicus* | Leech | NA | NA | NA | NA | NA | NA | NA | 8.23 | 17.7 | 46.4 | 56 | 21.6 | 38.2 |
| 31 | *Rattus norvegicus* | Vena | 3.93 | 2.65 | 0.49 | 0.79 | 67.4 | 12.47 | 20.2 | 5.62 | 11.1 | 31.6 | 56 | 19.8 | 35.1 |
| 32 | *Vicugna pacos* | Leech | 12.88 | 1.93 | 0.18 | 10.37 | 15.0 | 1.40 | 80.5 | 13.54 | 17.6 | 32.4 | 24 | 13.0 | 54.3 |
| 32 | *Vicugna pacos* | Vena | 9.51 | 0.99 | 0.05 | 7.70 | 10.4 | 0.53 | 80.9 | 11.86 | 14.8 | 28.0 | 24 | 12.5 | 52.8 |
| 33 | *Vicugna pacos* | Leech | 21.40 | 0.62 | 0.12 | 19.32 | 2.9 | 0.56 | 90.3 | 12.27 | 17.5 | 33.6 | 25 | 13.2 | 52.2 |
| 33 | *Vicugna pacos* | Vena | 18.29 | 1.38 | 0.12 | 15.05 | 7.6 | 0.66 | 82.3 | 11.60 | 14.9 | 29.1 | 25 | 12.8 | 51.2 |
| 34 | *Vicugna pacos* | Leech | 16.14 | 3.07 | 0.10 | 11.36 | 19.0 | 0.62 | 70.4 | 10.79 | 13.2 | 25.6 | 24 | 12.3 | 51.7 |
| 34 | *Vicugna pacos* | Vena | 13.95 | 2.06 | 0.10 | 9.16 | 14.8 | 0.72 | 65.7 | 10.89 | 13.5 | 25.3 | 23 | 12.4 | 53.4 |
| 35 | *Vicugna pacos* | Leech | 11.00 | 2.07 | 0.09 | 7.32 | 18.8 | 0.82 | 66.5 | 11.07 | 13.5 | 25.7 | 23 | 12.2 | 52.6 |
| 35 | *Vicugna pacos* | Vena | 9.86 | 1.97 | 0.06 | 6.18 | 20.0 | 0.61 | 62.7 | 10.96 | 13.5 | 25.1 | 23 | 12.3 | 54.0 |
| 36 | *Vicugna pacos* | Leech | 18.29 | 5.17 | 0.15 | 10.84 | 28.3 | 0.82 | 59.3 | 14.03 | 17.6 | 35.9 | 26 | 12.5 | 49.0 |
| 36 | *Vicugna pacos* | Vena | 12.73 | 4.30 | 0.06 | 7.55 | 33.8 | 0.47 | 59.3 | 14.63 | 18.3 | NA | NA | 12.5 | NA |
| 37 | *Vicugna pacos* | Leech | 23.54 | 7.88 | 0.12 | 14.45 | 33.5 | 0.51 | 61.4 | 17.13 | 21.7 | 42.6 | 25 | 12.7 | 50.9 |
| 37 | *Vicugna pacos* | Vena | 9.26 | 3.57 | 0.06 | 4.74 | 38.6 | 0.65 | 51.2 | 13.11 | 15.9 | 31.8 | 24 | 12.2 | 50.1 |
| 38 | *Vicugna pacos* | Leech | 17.18 | 1.92 | 0.13 | 13.19 | 11.2 | 0.76 | 76.8 | 15.19 | 17.8 | 34.9 | 23 | 11.7 | 50.9 |
| 38 | *Vicugna pacos* | Vena | 15.57 | 2.25 | 0.09 | 11.97 | 14.4 | 0.58 | 76.9 | 15.52 | 18.1 | 35.4 | 23 | 11.6 | 51.0 |
| 39 | *Oryctolagus cuniculus* | Leech | 18.74 | 6.72 | 1.01 | 11.01 | 35.9 | 5.39 | 58.8 | 5.84 | 12.3 | 34.5 | 59 | 21.1 | 35.7 |
| 39 | *Oryctolagus cuniculus* | Vena | 10.61 | 0.46 | 0.48 | 9.67 | 4.3 | 4.52 | 91.2 | 5.72 | 10.6 | 33.5 | 59 | 18.4 | 31.5 |
| 40 | *Oryctolagus cuniculus* | Leech | 6.09 | 4.37 | 0.04 | 1.69 | 71.7 | 0.66 | 27.7 | 6.61 | 11.9 | 36.5 | 55 | 18.0 | 32.6 |
| 40 | *Oryctolagus cuniculus* | Vena | 4.60 | 2.28 | 0.40 | 1.92 | 49.7 | 8.70 | 41.7 | 6.22 | 10.5 | 34.1 | 55 | 16.9 | 30.8 |
| 41 | *Oryctolagus cuniculus* | Leech | 13.05 | 3.30 | 1.54 | 8.22 | 25.3 | 11.80 | 62.9 | 6.26 | 12.0 | 37.4 | 60 | 19.2 | 32.2 |
| 41 | *Oryctolagus cuniculus* | Vena | 9.07 | 2.07 | 0.70 | 6.31 | 22.8 | 7.72 | 69.5 | 5.51 | 11.1 | 32.4 | 59 | 20.1 | 34.1 |
| 42 | *Oryctolagus cuniculus* | Leech | 24.10 | 1.48 | 2.61 | 20.00 | 6.2 | 10.83 | 83.0 | 5.84 | 11.3 | 33.9 | 58 | 19.3 | 33.2 |
| 42 | *Oryctolagus cuniculus* | Vena | 12.66 | 2.51 | 0.90 | 9.25 | 19.8 | 7.11 | 73.1 | 5.04 | 9.8 | 29.3 | 58 | 19.5 | 33.6 |
| 43 | *Oryctolagus cuniculus* | Leech | 10.79 | 2.64 | 1.32 | 6.83 | 24.5 | 12.23 | 63.3 | 6.08 | 12.8 | 37.8 | 62 | 21.1 | 33.9 |
| 43 | *Oryctolagus cuniculus* | Vena | 4.83 | 0.59 | 0.50 | 3.74 | 12.2 | 10.35 | 77.4 | 5.38 | 10.6 | 33.5 | 62 | 19.7 | 31.6 |
| 44 | *Oryctolagus cuniculus* | Leech | 15.04 | 7.03 | 1.26 | 6.75 | 46.8 | 8.38 | 44.9 | 6.19 | 11.7 | 35.9 | 58 | 18.9 | 32.5 |
| 44 | *Oryctolagus cuniculus* | Vena | 5.53 | 0.77 | 0.60 | 4.17 | 13.9 | 10.85 | 75.3 | 5.33 | 10.8 | 31.8 | 60 | 20.3 | 34.1 |
| 45 | *Oryctolagus cuniculus* | Leech | 16.40 | 10.19 | 1.89 | 4.85 | 60.2 | 11.52 | 28.6 | 6.35 | 11.8 | 35.3 | 56 | 18.6 | 33.4 |
| 45 | *Oryctolagus cuniculus* | Vena | 9.06 | 3.16 | 0.19 | 5.70 | 34.9 | 2.10 | 62.9 | 5.76 | 10.8 | 31.9 | 55 | 18.7 | 33.8 |
| 46 | *Macropus rufogriseus* | Leech | 22.74 | 9.11 | 0.13 | 5.66 | 40.0 | 0.57 | 24.9 | 5.35 | 19.1 | 43.3 | 81 | 35.6 | 44.1 |
| 46 | *Macropus rufogriseus* | Vena | 16.53 | 1.74 | 0.11 | 14.32 | 10.5 | 0.67 | 86.6 | 6.53 | 20.8 | 57.4 | 88 | 31.9 | 36.3 |
| 47 | *Dolichotis patagonum* | Leech | 4.78 | 1.25 | 0.04 | 3.49 | 26.1 | 0.84 | 73.0 | 8.90 | 21.2 | 58.6 | 66 | 23.9 | 36.2 |
| 47 | *Dolichotis patagonum* | Vena | 4.17 | 1.20 | 0.51 | 2.46 | 28.7 | 12.23 | 59.0 | 9.06 | 21.5 | 60.3 | 67 | 23.7 | 35.7 |
| 48 | *Oryctolagus cuniculus* | Leech | 10.74 | 2.03 | 0.68 | 8.03 | 18.9 | 6.33 | 74.8 | 5.06 | 10.2 | 29.9 | 59 | 20.1 | 34.0 |
| 48 | *Oryctolagus cuniculus* | Vena | 9.43 | 1.59 | 0.51 | 7.34 | 16.9 | 5.41 | 77.8 | 5.04 | 9.9 | 29.9 | 59 | 19.6 | 33.1 |
| 49 | *Oryctolagus cuniculus* | Leech | 24.96 | 0.08 | 1.20 | 23.68 | 0.3 | 4.81 | 94.9 | 4.40 | 8.3 | 23.2 | 57 | 19.0 | 33.2 |
| 49 | *Oryctolagus cuniculus* | Vena | 21.72 | 0.22 | 0.99 | 20.51 | 1.0 | 4.56 | 94.4 | 3.69 | 8.2 | 21.5 | 58 | 22.2 | 38.0 |
| 50 | *Oryctolagus cuniculus* | Leech | 14.16 | 5.14 | 0.75 | 8.28 | 36.3 | 5.30 | 58.4 | 5.34 | 9.7 | 30.0 | 56 | 18.2 | 32.4 |
| 50 | *Oryctolagus cuniculus* | Vena | 11.56 | 1.21 | 0.64 | 9.72 | 10.4 | 5.54 | 84.1 | 5.28 | 9.9 | 29.8 | 57 | 18.7 | 33.1 |
| 51 | *Oryctolagus cuniculus* | Leech | 17.04 | 4.21 | 1.96 | 10.86 | 24.7 | 11.50 | 63.7 | 6.32 | 12.3 | 35.7 | 57 | 19.5 | 34.5 |
| 51 | *Oryctolagus cuniculus* | Vena | 8.24 | 0.07 | 0.88 | 7.30 | 0.9 | 10.68 | 88.5 | 6.10 | 11.8 | 34.8 | 57 | 19.3 | 33.8 |
| 52 | *Oryctolagus cuniculus* | Leech | 15.55 | 5.56 | 0.11 | 9.87 | 35.8 | 0.71 | 63.5 | 6.19 | 12.5 | 35.4 | 57 | 20.3 | 35.4 |
| 52 | *Oryctolagus cuniculus* | Vena | 6.26 | 0.22 | 0.52 | 5.52 | 3.6 | 8.31 | 88.1 | 5.59 | 10.7 | 32.1 | 57 | 19.1 | 33.3 |
| 53 | *Oryctolagus cuniculus* | Leech | 15.14 | 5.18 | 1.02 | 8.94 | 34.2 | 6.74 | 59.1 | 4.26 | 11.2 | 25.4 | 60 | 26.2 | 44.0 |
| 53 | *Oryctolagus cuniculus* | Vena | 7.52 | 0.71 | 0.34 | 6.47 | 9.5 | 4.52 | 86.0 | 5.75 | 11.3 | 34.5 | 60 | 19.7 | 32.7 |
| 54 | *Oryctolagus cuniculus* | Leech | 19.14 | 3.64 | 1.87 | 13.63 | 19.0 | 9.77 | 71.2 | 6.19 | 12.1 | 35.8 | 58 | 19.5 | 33.7 |
| 54 | *Oryctolagus cuniculus* | Vena | 10.75 | 1.91 | 0.82 | 8.02 | 17.8 | 7.63 | 74.6 | 5.88 | 11.2 | 34.3 | 58 | 19.0 | 32.6 |
| 55 | *Oryctolagus cuniculus* | Leech | 10.23 | 1.09 | 0.98 | 8.15 | 10.7 | 9.58 | 79.7 | 5.62 | 11.4 | 35.1 | 62 | 20.3 | 32.5 |
| 55 | *Oryctolagus cuniculus* | Vena | 6.90 | 1.07 | 0.45 | 5.38 | 15.5 | 6.52 | 78.0 | 4.88 | 10.5 | 30.3 | 62 | 21.5 | 34.5 |
| 56 | *Oryctolagus cuniculus* | Leech | 15.39 | 8.54 | 1.10 | 5.74 | 55.5 | 7.15 | 37.3 | 7.35 | 12.3 | 46.6 | 63 | 16.7 | 26.4 |
| 56 | *Oryctolagus cuniculus* | Vena | 6.52 | 1.12 | 0.44 | 4.97 | 17.1 | 6.75 | 76.2 | 8.70 | 9.9 | 55.6 | 64 | 11.4 | 17.9 |
| 57 | *Oryctolagus cuniculus* | Leech | 9.49 | 2.39 | 0.63 | 6.48 | 25.1 | 6.64 | 68.2 | 6.51 | 12.2 | 41.9 | 64 | 18.7 | 29.0 |
| 57 | *Oryctolagus cuniculus* | Vena | 6.16 | 0.09 | 0.31 | 5.76 | 1.4 | 5.03 | 93.5 | 6.75 | 11.9 | 43.4 | 64 | 17.6 | 27.4 |
| 58 | *Oryctolagus cuniculus* | Leech | 16.11 | 5.59 | 0.81 | 9.71 | 34.7 | 5.03 | 60.3 | 6.68 | 12.6 | 38.5 | 58 | 18.8 | 32.7 |
| 58 | *Oryctolagus cuniculus* | Vena | 11.88 | 2.82 | 0.63 | 8.44 | 23.7 | 5.30 | 71.0 | 6.60 | 12.9 | 39.9 | 61 | 19.5 | 32.3 |
| 59 | *Oryctolagus cuniculus* | Leech | 15.45 | 6.50 | 0.74 | 8.21 | 42.1 | 4.79 | 53.1 | 5.81 | 12.0 | 36.6 | 63 | 20.6 | 32.7 |
| 59 | *Oryctolagus cuniculus* | Vena | 5.98 | 2.81 | 0.04 | 3.12 | 47.1 | 0.67 | 52.2 | 4.70 | 9.4 | 29.3 | 62 | 20.0 | 32.2 |
| 60 | *Oryctolagus cuniculus* | Leech | 18.34 | 5.03 | 1.52 | 11.79 | 27.4 | 8.29 | 64.3 | 5.37 | 11.0 | 33.7 | 63 | 20.5 | 32.7 |
| 60 | *Oryctolagus cuniculus* | Vena | 6.57 | 1.92 | 0.05 | 4.60 | 29.2 | 0.76 | 70.1 | 4.57 | 9.7 | 28.8 | 63 | 21.2 | 33.7 |
| 61 | *Oryctolagus cuniculus* | Leech | 25.75 | 0.26 | 2.15 | 23.35 | 1.0 | 8.35 | 90.7 | 5.19 | 9.8 | 29.1 | 56 | 18.9 | 33.8 |
| 61 | *Oryctolagus cuniculus* | Vena | 11.20 | 0.02 | 0.47 | 10.71 | 0.2 | 4.20 | 95.6 | 4.87 | 9.3 | 27.4 | 56 | 19.0 | 33.8 |
| 62 | *Oryctolagus cuniculus* | Leech | 10.39 | 1.85 | 1.44 | 7.09 | 17.8 | 13.86 | 68.3 | 6.07 | 12.1 | 36.5 | 60 | 19.9 | 33.1 |
| 62 | *Oryctolagus cuniculus* | Vena | 4.09 | 0.47 | 0.52 | 3.10 | 11.4 | 12.71 | 75.9 | 5.03 | 10.0 | 30.0 | 60 | 19.8 | 33.2 |
| 63 | *Sus scrofa* | Leech | 15.93 | 4.61 | 1.01 | 10.31 | 28.9 | 6.34 | 64.7 | 8.32 | 13.6 | 38.3 | 46 | 16.3 | 35.4 |
| 63 | *Sus scrofa* | Vena | 15.72 | 1.65 | 1.30 | 12.76 | 10.5 | 8.27 | 81.2 | 8.18 | 13.1 | 37.5 | 46 | 16.0 | 34.9 |
| 64 | *Capra ibex* | Leech | 13.15 | 3.51 | 0.07 | 9.57 | 26.7 | 0.53 | 72.8 | 15.40 | 14.7 | 34.8 | 23 | 9.6 | 42.4 |
| 64 | *Capra ibex* | Vena | 11.38 | 1.75 | 0.06 | 9.57 | 15.4 | 0.53 | 84.0 | 14.68 | 12.7 | 34.4 | 23 | 8.6 | 36.8 |
| 65 | *Capra ibex* | Leech | 8.88 | 1.99 | 0.06 | 6.83 | 7.5 | 0.68 | 76.9 | 17.67 | 13.9 | 40.6 | 23 | 7.9 | 34.2 |
| 65 | *Capra ibex* | Vena | 11.54 | 0.86 | 0.06 | 10.61 | 7.5 | 0.52 | 92.0 | 17.82 | 13.3 | 39.4 | 22 | 7.5 | 33.8 |
| 66 | *Capra ibex* | Leech | 3.74 | 1.00 | 0.02 | 2.72 | 26.7 | 0.53 | 72.7 | NA | NA | NA | 22 | 7.8 | 36.0 |
| 66 | *Capra ibex* | Vena | 5.26 | 0.61 | 0.03 | 4.62 | 11.6 | 0.57 | 87.8 | 18.20 | 14.3 | 40.4 | 22 | 7.9 | 35.4 |
| 67 | *Vicugna pacos* | Leech | 20.37 | 6.87 | 0.13 | 11.69 | 33.7 | 0.64 | 57.4 | 15.56 | 22.2 | 38.6 | 25 | 14.3 | 57.6 |
| 67 | *Vicugna pacos* | Vena | 9.14 | 1.90 | 0.06 | 6.00 | 20.8 | 0.66 | 65.7 | 12.42 | 15.4 | 30.4 | 24 | 12.4 | 50.9 |

**Supplementary Figure 1** Distributions of values obtained using venipuncture, leeches, and leeches with corrections of the leech-induced parameter alterations. Corrected values (*L_c_*_)_ were obtained using a regression formula *L_c_* = *a* + *b* × *L*, where *L* are blood parameter values measured in leech-derived blood samples, and *a* and *b* are the intercept and slope coefficients, respectively, from the regression model of venipuncture-derived values (*V*) on leech-derived values. Species reference values are shaded in grey.


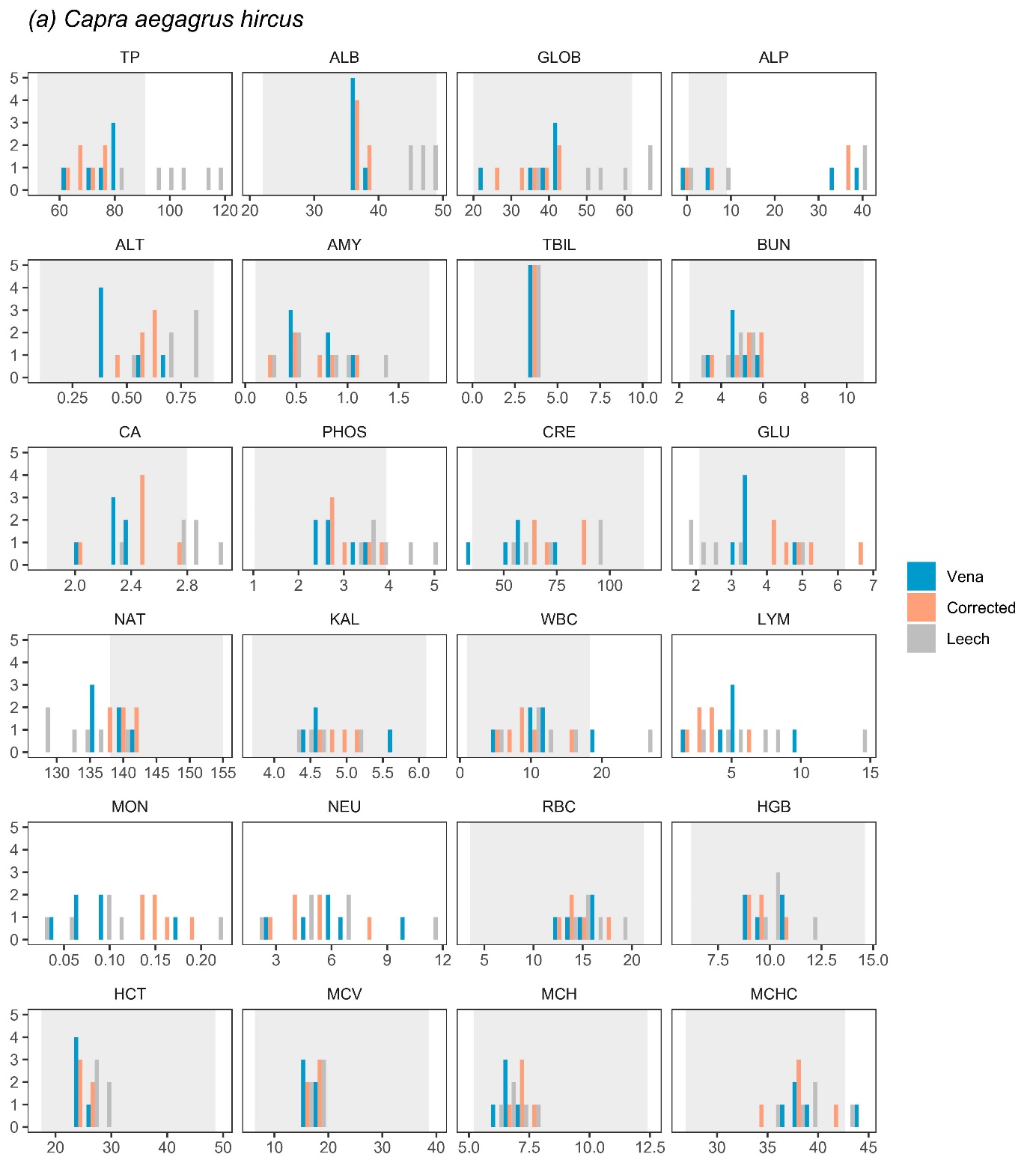


Total protein (TP, g/L), albumin (ALB, g/L), globulin (GLOB, g/L), alkaline phosphatase (ALP, µkat/L), alanine aminotrasferase (ALT, µkat/L), amylase (AMY, µkat/L), total bilirubin (TBIL, µmol/L), blood urea nitrogen (BUN, mmol/L), calcium (CA, mmol/L), phosphorus (PHOS, mmol/L), creatinine (CRE, µmol/L), glucose (GLU, mmol/L), sodium (NAT, mmol/L), potassium (KAL, mmol/L),white blood cell count (WBC, 10^9^/L), lymphocyte count (LYM, 10^9^/L ), monocyte count (MON, 10^9^/L), neutrophil count (NEU, 10^9^/L), red blood cell count (RBC, 10^12^/L), haemoglobin (HGB, g/dl), haematocrit (HCT, %), mean cell volume (MCV, fL), mean corpuscular haemoglobin (MCH, pg), and mean corpuscular haemoglobin concentration (MCHC, g/dL).


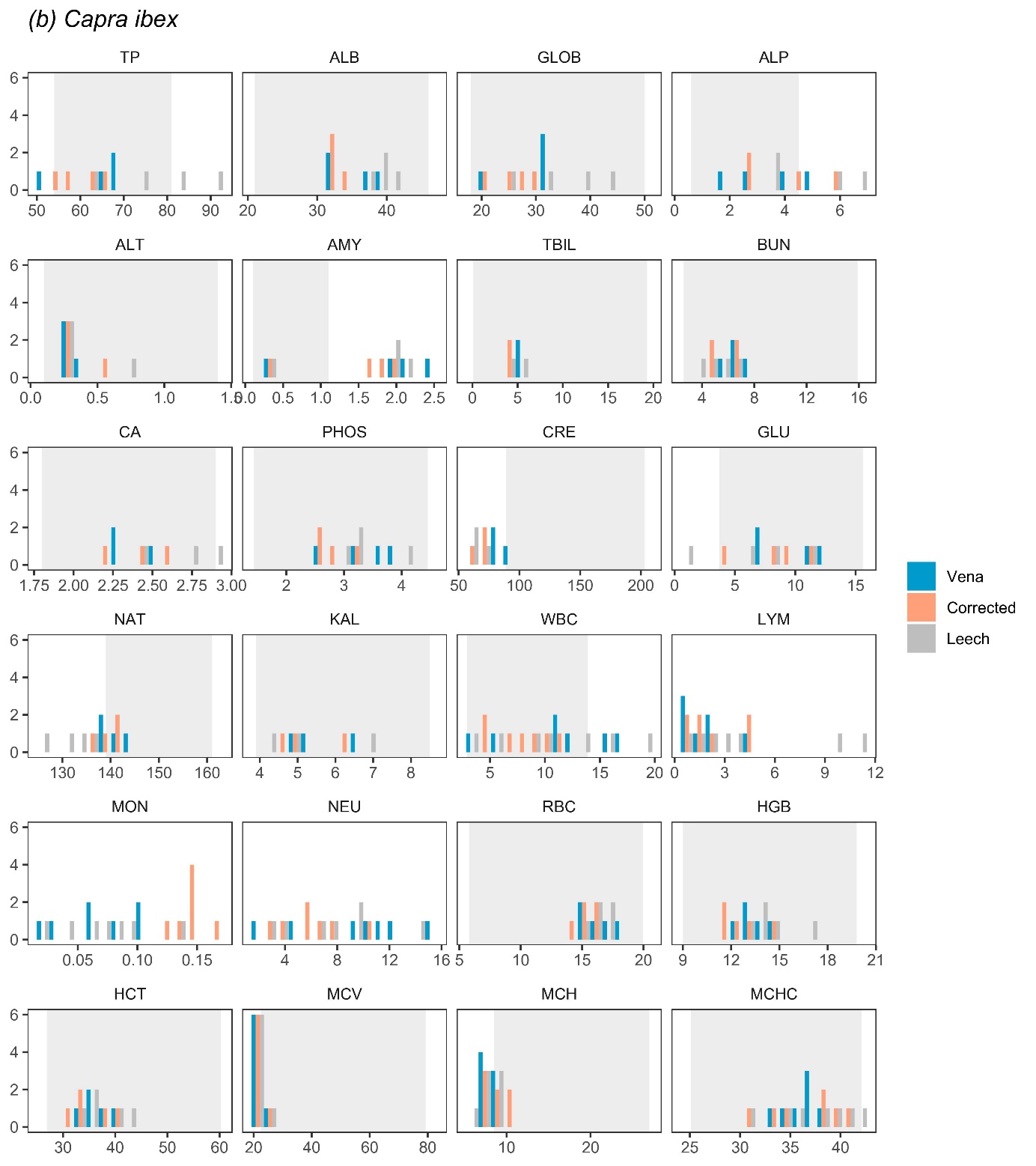


Total protein (TP, g/L), albumin (ALB, g/L), globulin (GLOB, g/L), alkaline phosphatase (ALP, µkat/L), alanine aminotrasferase (ALT, µkat/L), amylase (AMY, µkat/L), total bilirubin (TBIL, µmol/L), blood urea nitrogen (BUN, mmol/L), calcium (CA, mmol/L), phosphorus (PHOS, mmol/L), creatinine (CRE, µmol/L), glucose (GLU, mmol/L), sodium (NAT, mmol/L), potassium (KAL, mmol/L),white blood cell count (WBC, 10^9^/L), lymphocyte count (LYM, 10^9^/L ), monocyte count (MON, 10^9^/L), neutrophil count (NEU, 10^9^/L), red blood cell count (RBC, 10^12^/L), haemoglobin (HGB, g/dl), haematocrit (HCT, %), mean cell volume (MCV, fL), mean corpuscular haemoglobin (MCH, pg), and mean corpuscular haemoglobin concentration (MCHC, g/dL).


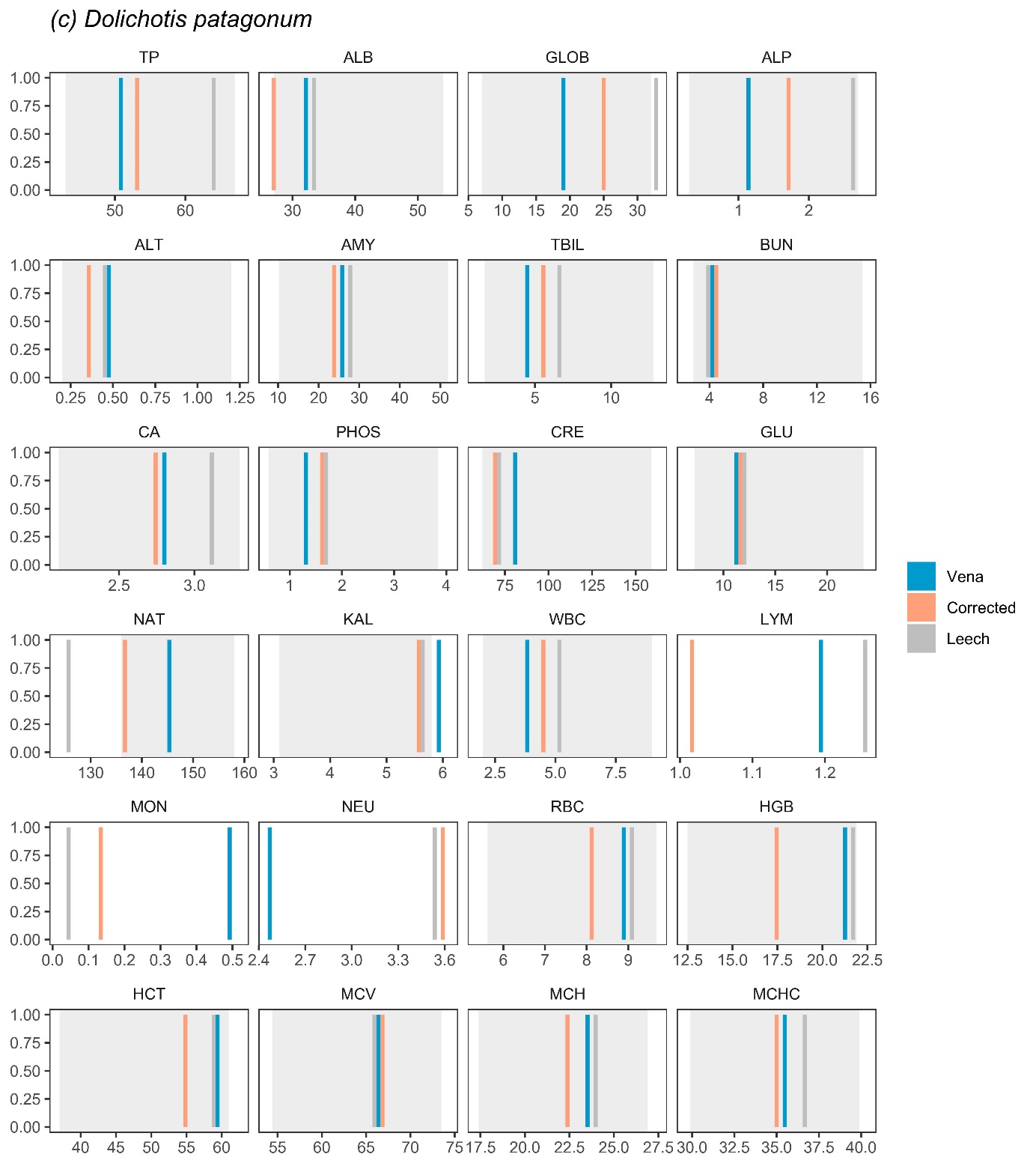


Total protein (TP, g/L), albumin (ALB, g/L), globulin (GLOB, g/L), alkaline phosphatase (ALP, µkat/L), alanine aminotrasferase (ALT, µkat/L), amylase (AMY, µkat/L), total bilirubin (TBIL, µmol/L), blood urea nitrogen (BUN, mmol/L), calcium (CA, mmol/L), phosphorus (PHOS, mmol/L), creatinine (CRE, µmol/L), glucose (GLU, mmol/L), sodium (NAT, mmol/L), potassium (KAL, mmol/L),white blood cell count (WBC, 10^9^/L), lymphocyte count (LYM, 10^9^/L ), monocyte count (MON, 10^9^/L), neutrophil count (NEU, 10^9^/L), red blood cell count (RBC, 10^12^/L), haemoglobin (HGB, g/dl), haematocrit (HCT, %), mean cell volume (MCV, fL), mean corpuscular haemoglobin (MCH, pg), and mean corpuscular haemoglobin concentration (MCHC, g/dL).


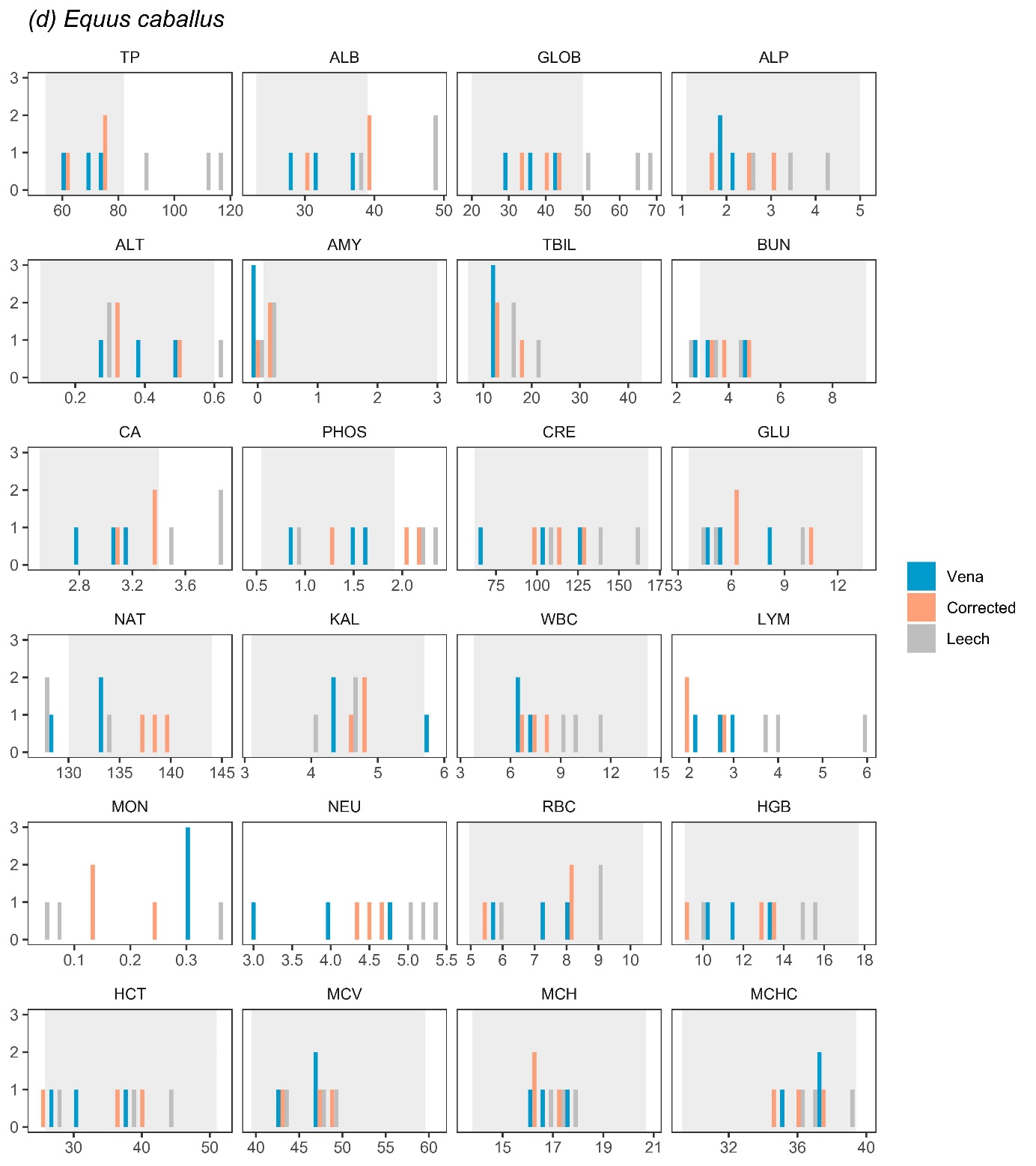


Total protein (TP, g/L), albumin (ALB, g/L), globulin (GLOB, g/L), alkaline phosphatase (ALP, µkat/L), alanine aminotrasferase (ALT, µkat/L), amylase (AMY, µkat/L), total bilirubin (TBIL, µmol/L), blood urea nitrogen (BUN, mmol/L), calcium (CA, mmol/L), phosphorus (PHOS, mmol/L), creatinine (CRE, µmol/L), glucose (GLU, mmol/L), sodium (NAT, mmol/L), potassium (KAL, mmol/L),white blood cell count (WBC, 10^9^/L), lymphocyte count (LYM, 10^9^/L ), monocyte count (MON, 10^9^/L), neutrophil count (NEU, 10^9^/L), red blood cell count (RBC, 10^12^/L), haemoglobin (HGB, g/dl), haematocrit (HCT, %), mean cell volume (MCV, fL), mean corpuscular haemoglobin (MCH, pg), and mean corpuscular haemoglobin concentration (MCHC, g/dL).


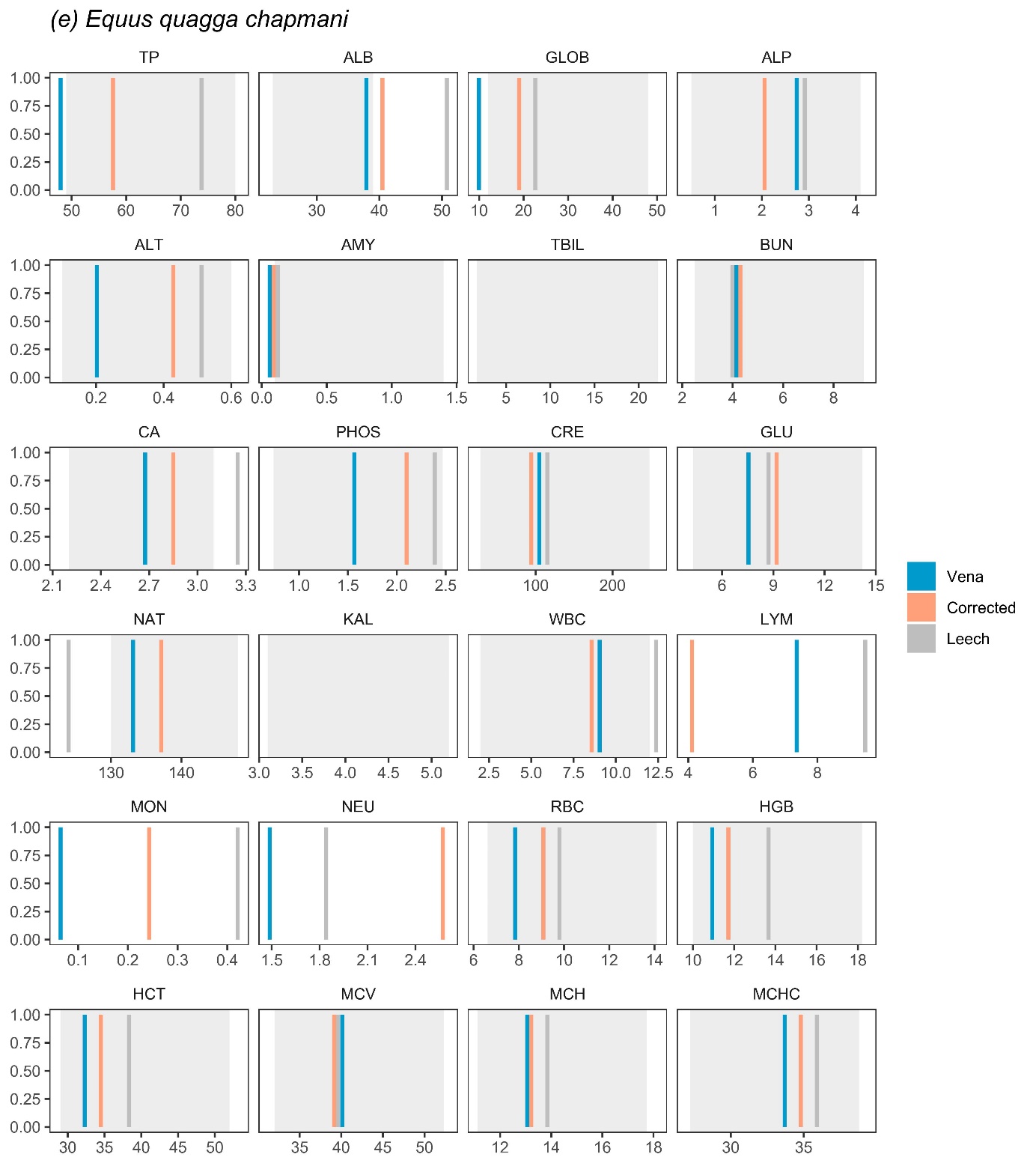


Total protein (TP, g/L), albumin (ALB, g/L), globulin (GLOB, g/L), alkaline phosphatase (ALP, µkat/L), alanine aminotrasferase (ALT, µkat/L), amylase (AMY, µkat/L), total bilirubin (TBIL, µmol/L), blood urea nitrogen (BUN, mmol/L), calcium (CA, mmol/L), phosphorus (PHOS, mmol/L), creatinine (CRE, µmol/L), glucose (GLU, mmol/L), sodium (NAT, mmol/L), potassium (KAL, mmol/L),white blood cell count (WBC, 10^9^/L), lymphocyte count (LYM, 10^9^/L ), monocyte count (MON, 10^9^/L), neutrophil count (NEU, 10^9^/L), red blood cell count (RBC, 10^12^/L), haemoglobin (HGB, g/dl), haematocrit (HCT, %), mean cell volume (MCV, fL), mean corpuscular haemoglobin (MCH, pg), and mean corpuscular haemoglobin concentration (MCHC, g/dL).


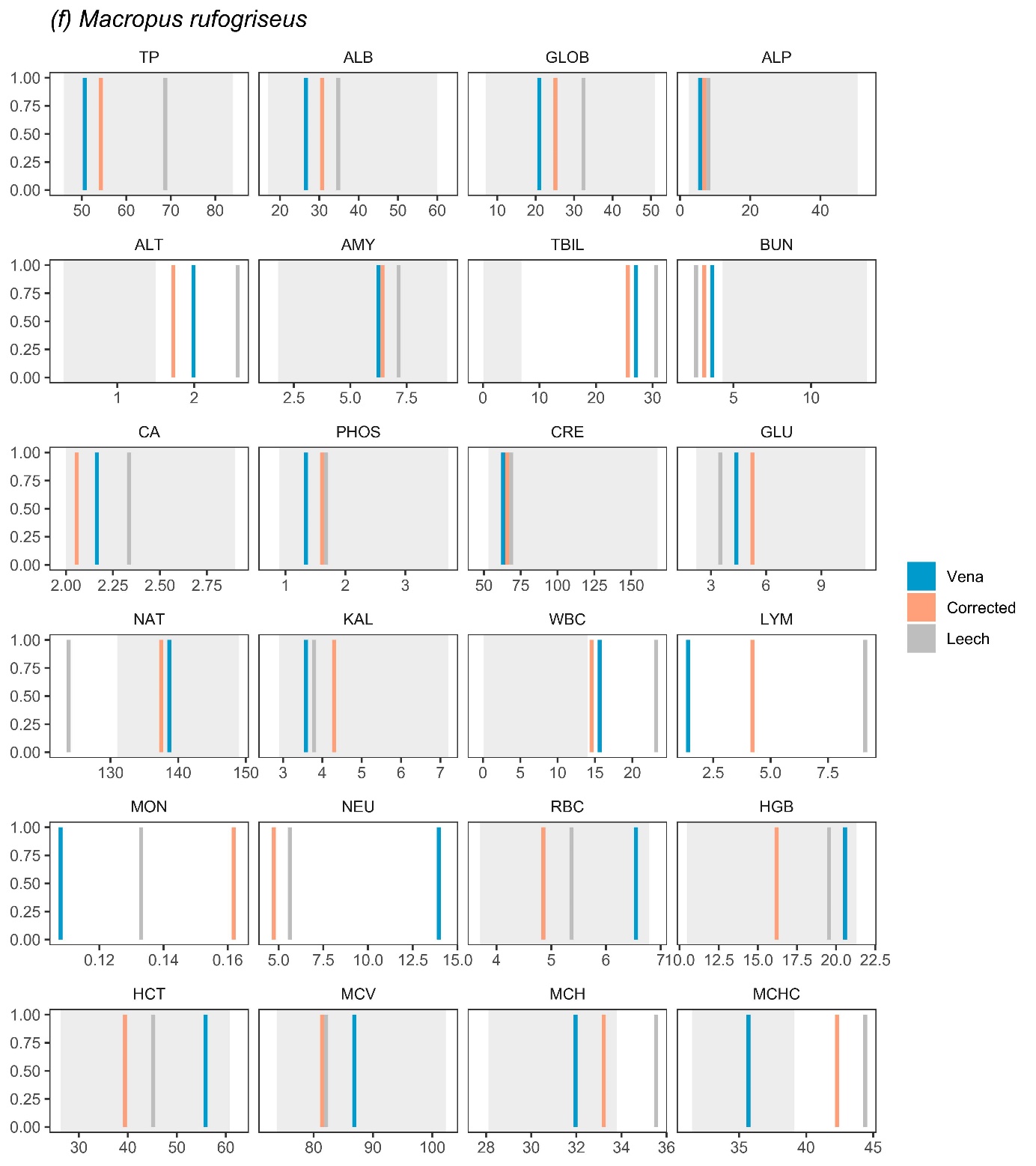


Total protein (TP, g/L), albumin (ALB, g/L), globulin (GLOB, g/L), alkaline phosphatase (ALP, µkat/L), alanine aminotrasferase (ALT, µkat/L), amylase (AMY, µkat/L), total bilirubin (TBIL, µmol/L), blood urea nitrogen (BUN, mmol/L), calcium (CA, mmol/L), phosphorus (PHOS, mmol/L), creatinine (CRE, µmol/L), glucose (GLU, mmol/L), sodium (NAT, mmol/L), potassium (KAL, mmol/L),white blood cell count (WBC, 10^9^/L), lymphocyte count (LYM, 10^9^/L ), monocyte count (MON, 10^9^/L), neutrophil count (NEU, 10^9^/L), red blood cell count (RBC, 10^12^/L), haemoglobin (HGB, g/dl), haematocrit (HCT, %), mean cell volume (MCV, fL), mean corpuscular haemoglobin (MCH, pg), and mean corpuscular haemoglobin concentration (MCHC, g/dL).


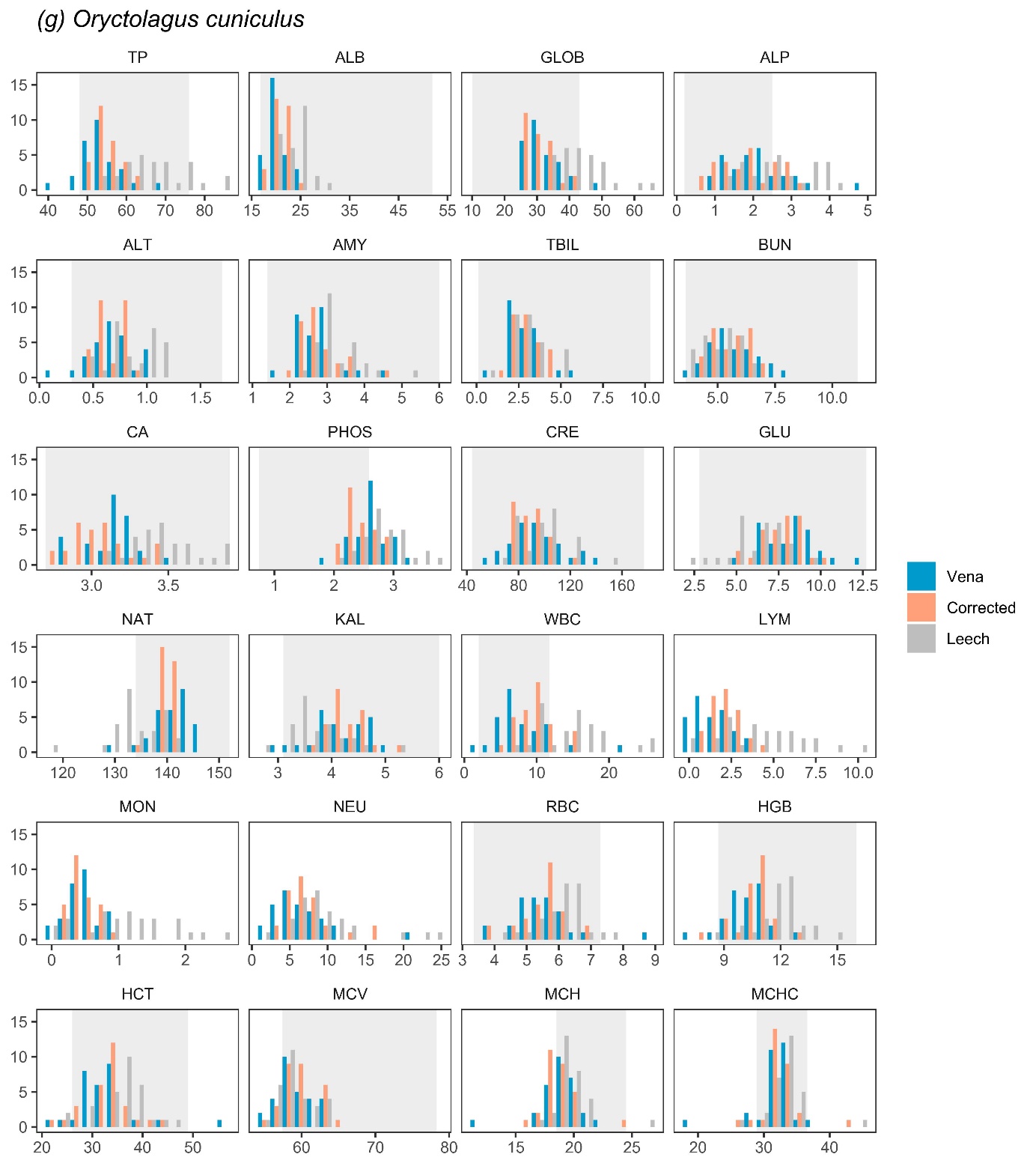


Total protein (TP, g/L), albumin (ALB, g/L), globulin (GLOB, g/L), alkaline phosphatase (ALP, µkat/L), alanine aminotrasferase (ALT, µkat/L), amylase (AMY, µkat/L), total bilirubin (TBIL, µmol/L), blood urea nitrogen (BUN, mmol/L), calcium (CA, mmol/L), phosphorus (PHOS, mmol/L), creatinine (CRE, µmol/L), glucose (GLU, mmol/L), sodium (NAT, mmol/L), potassium (KAL, mmol/L),white blood cell count (WBC, 10^9^/L), lymphocyte count (LYM, 10^9^/L ), monocyte count (MON, 10^9^/L), neutrophil count (NEU, 10^9^/L), red blood cell count (RBC, 10^12^/L), haemoglobin (HGB, g/dl), haematocrit (HCT, %), mean cell volume (MCV, fL), mean corpuscular haemoglobin (MCH, pg), and mean corpuscular haemoglobin concentration (MCHC, g/dL).


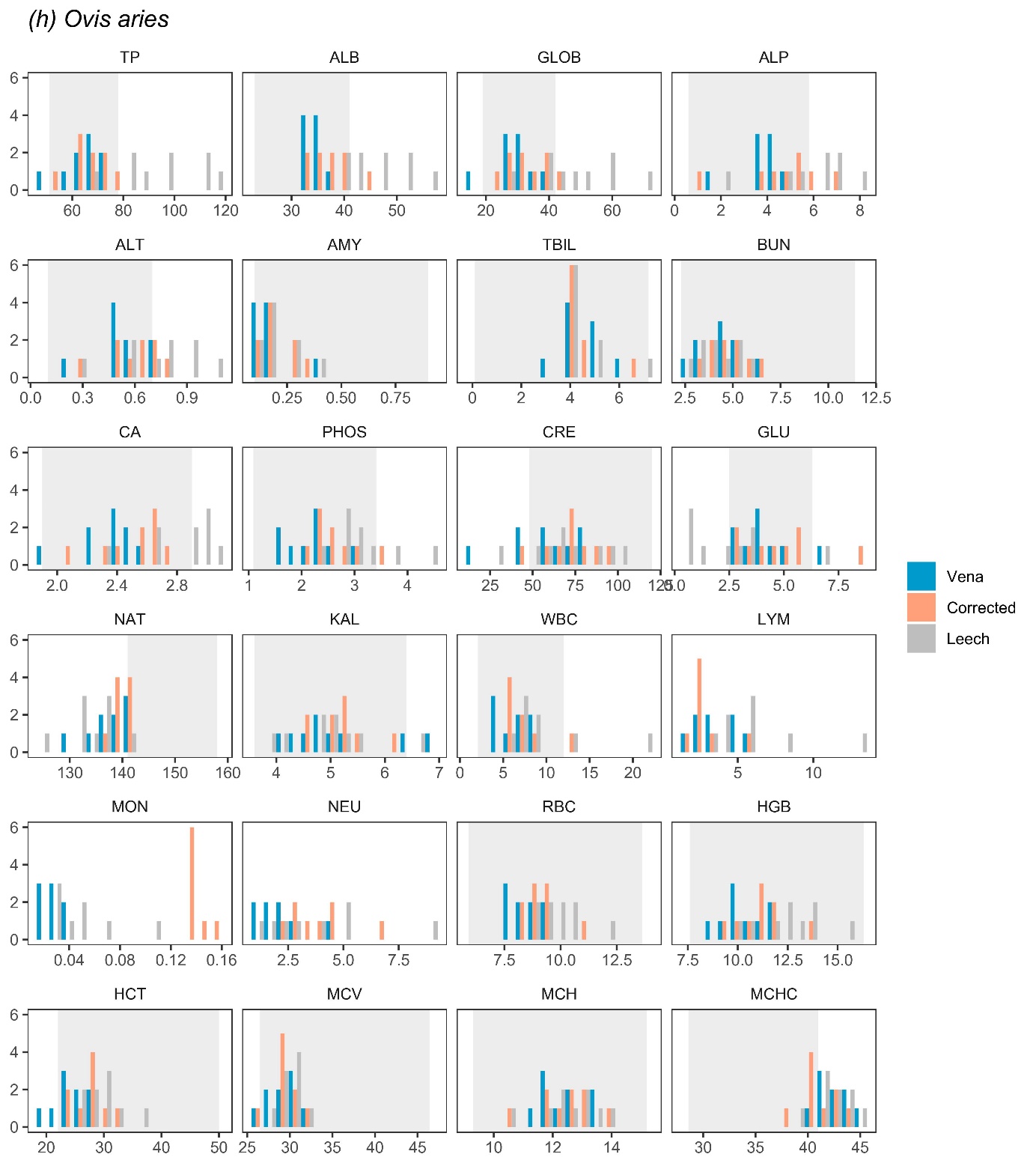


Total protein (TP, g/L), albumin (ALB, g/L), globulin (GLOB, g/L), alkaline phosphatase (ALP, µkat/L), alanine aminotrasferase (ALT, µkat/L), amylase (AMY, µkat/L), total bilirubin (TBIL, µmol/L), blood urea nitrogen (BUN, mmol/L), calcium (CA, mmol/L), phosphorus (PHOS, mmol/L), creatinine (CRE, µmol/L), glucose (GLU, mmol/L), sodium (NAT, mmol/L), potassium (KAL, mmol/L),white blood cell count (WBC, 10^9^/L), lymphocyte count (LYM, 10^9^/L ), monocyte count (MON, 10^9^/L), neutrophil count (NEU, 10^9^/L), red blood cell count (RBC, 10^12^/L), haemoglobin (HGB, g/dl), haematocrit (HCT, %), mean cell volume (MCV, fL), mean corpuscular haemoglobin (MCH, pg), and mean corpuscular haemoglobin concentration (MCHC, g/dL).


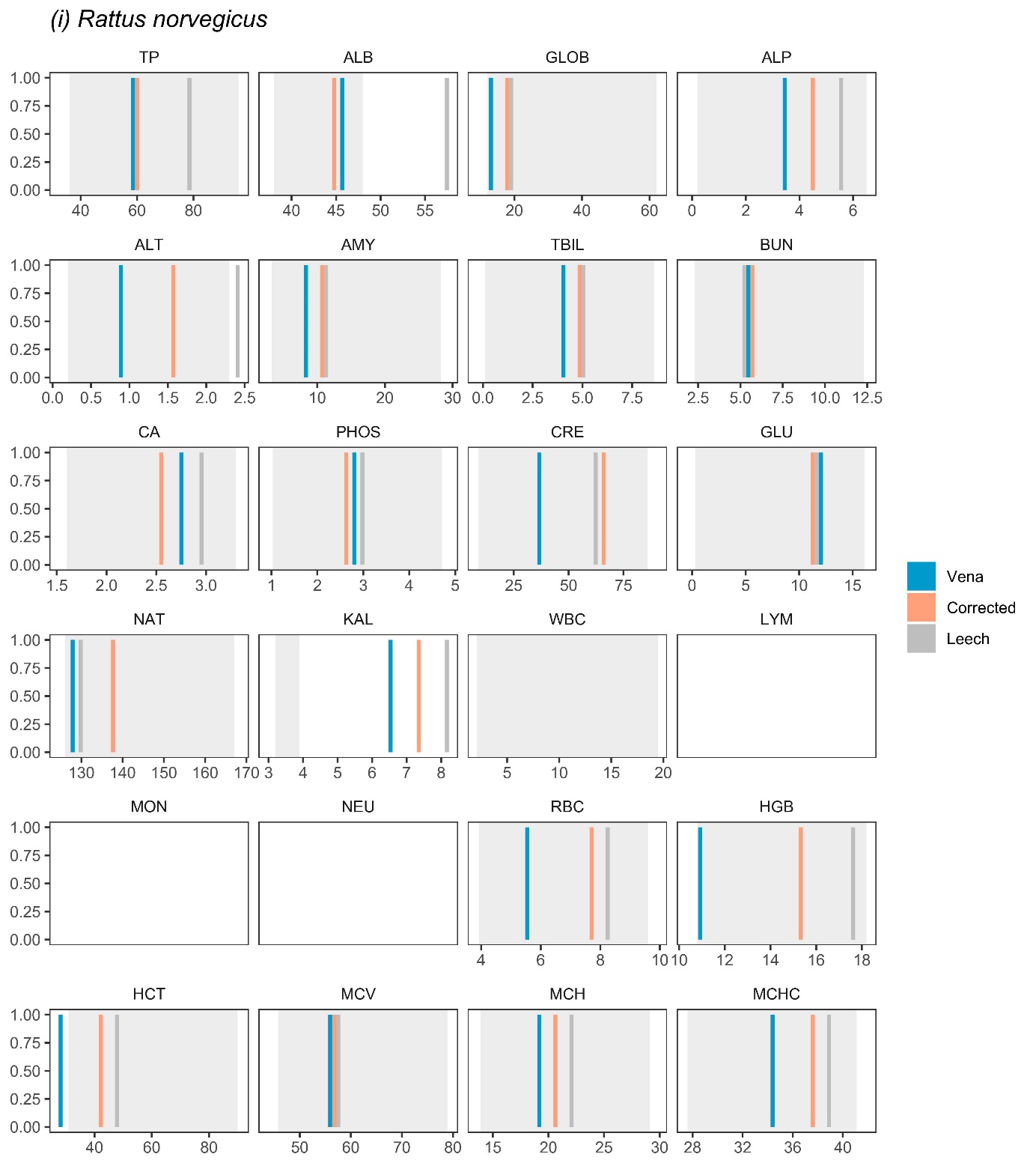


Total protein (TP, g/L), albumin (ALB, g/L), globulin (GLOB, g/L), alkaline phosphatase (ALP, µkat/L), alanine aminotrasferase (ALT, µkat/L), amylase (AMY, µkat/L), total bilirubin (TBIL, µmol/L), blood urea nitrogen (BUN, mmol/L), calcium (CA, mmol/L), phosphorus (PHOS, mmol/L), creatinine (CRE, µmol/L), glucose (GLU, mmol/L), sodium (NAT, mmol/L), potassium (KAL, mmol/L),white blood cell count (WBC, 10^9^/L), lymphocyte count (LYM, 10^9^/L ), monocyte count (MON, 10^9^/L), neutrophil count (NEU, 10^9^/L), red blood cell count (RBC, 10^12^/L), haemoglobin (HGB, g/dl), haematocrit (HCT, %), mean cell volume (MCV, fL), mean corpuscular haemoglobin (MCH, pg), and mean corpuscular haemoglobin concentration (MCHC, g/dL).


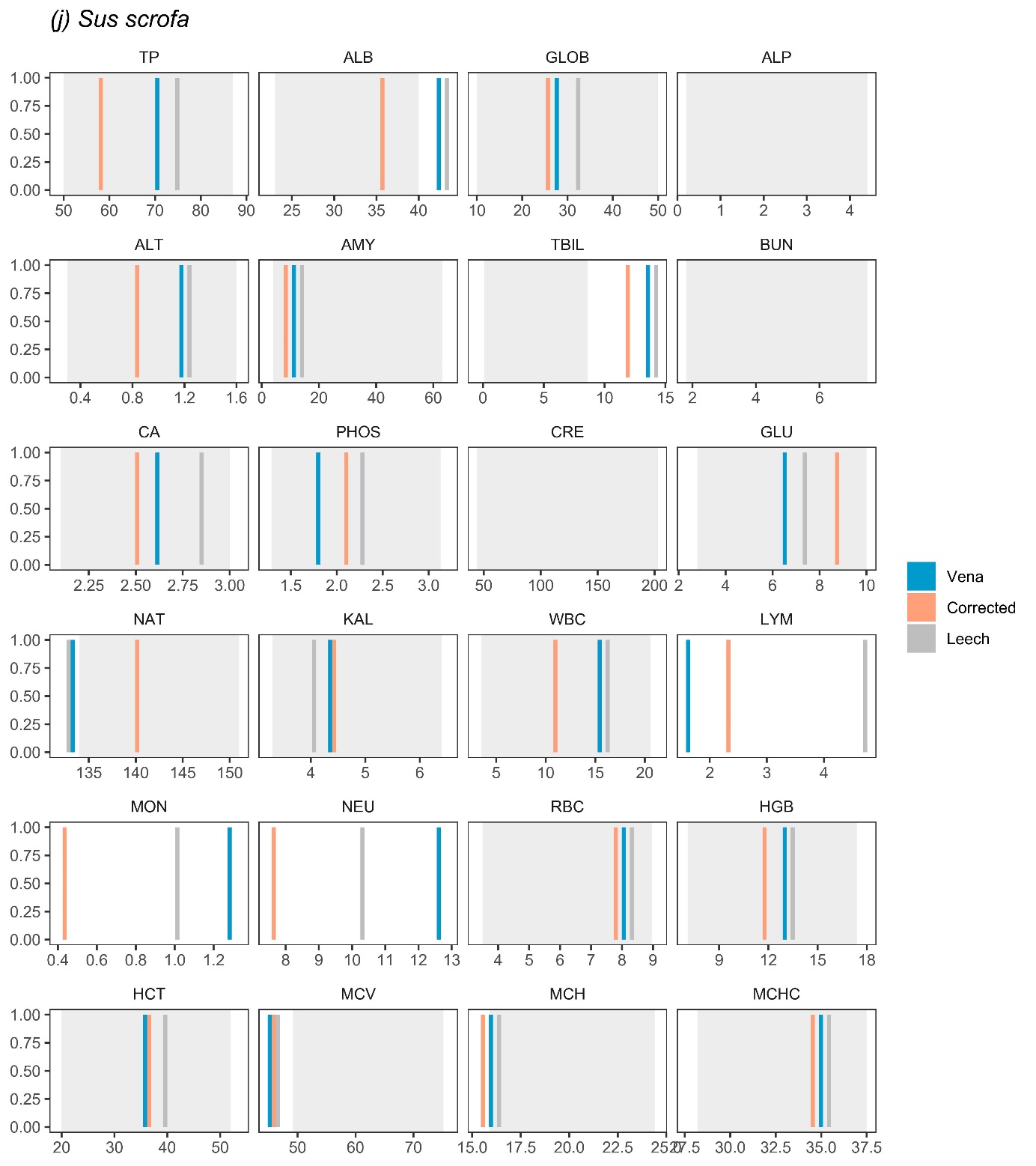


Total protein (TP, g/L), albumin (ALB, g/L), globulin (GLOB, g/L), alkaline phosphatase (ALP, µkat/L), alanine aminotrasferase (ALT, µkat/L), amylase (AMY, µkat/L), total bilirubin (TBIL, µmol/L), blood urea nitrogen (BUN, mmol/L), calcium (CA, mmol/L), phosphorus (PHOS, mmol/L), creatinine (CRE, µmol/L), glucose (GLU, mmol/L), sodium (NAT, mmol/L), potassium (KAL, mmol/L),white blood cell count (WBC, 10^9^/L), lymphocyte count (LYM, 10^9^/L ), monocyte count (MON, 10^9^/L), neutrophil count (NEU, 10^9^/L), red blood cell count (RBC, 10^12^/L), haemoglobin (HGB, g/dl), haematocrit (HCT, %), mean cell volume (MCV, fL), mean corpuscular haemoglobin (MCH, pg), and mean corpuscular haemoglobin concentration (MCHC, g/dL).


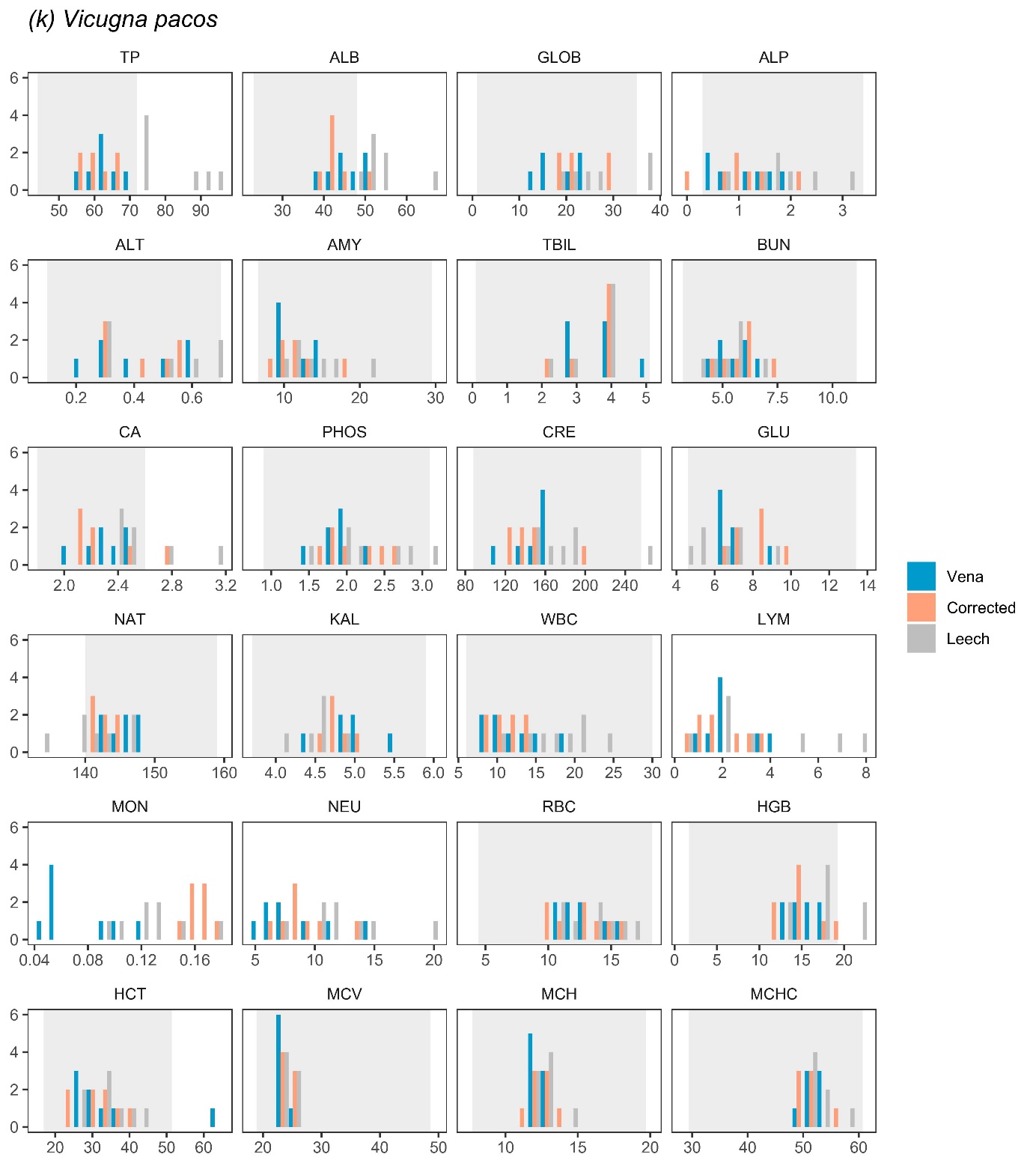


Total protein (TP, g/L), albumin (ALB, g/L), globulin (GLOB, g/L), alkaline phosphatase (ALP, µkat/L), alanine aminotrasferase (ALT, µkat/L), amylase (AMY, µkat/L), total bilirubin (TBIL, µmol/L), blood urea nitrogen (BUN, mmol/L), calcium (CA, mmol/L), phosphorus (PHOS, mmol/L), creatinine (CRE, µmol/L), glucose (GLU, mmol/L), sodium (NAT, mmol/L), potassium (KAL, mmol/L),white blood cell count (WBC, 10^9^/L), lymphocyte count (LYM, 10^9^/L ), monocyte count (MON, 10^9^/L), neutrophil count (NEU, 10^9^/L), red blood cell count (RBC, 10^12^/L), haemoglobin (HGB, g/dl), haematocrit (HCT, %), mean cell volume (MCV, fL), mean corpuscular haemoglobin (MCH, pg), and mean corpuscular haemoglobin concentration (MCHC, g/dL).
